# Detecting Selection in the HIV-1 Genome during Sexual Transmission Events

**DOI:** 10.3390/v14020406

**Published:** 2022-02-16

**Authors:** David Seifert, Beda Joos, Dominique L. Braun, Corinna S. Oberle, Corinne D. Schenkel, Herbert Kuster, Christina Grube, Jürg Böni, Sabine Yerly, Vincent Aubert, Thomas Klimkait, Huldrych F. Günthard, Niko Beerenwinkel, Karin J. Metzner, on behalf of the Swiss HIV Cohort Study

**Affiliations:** 1 Department of Biosystems Science and Engineering, ETH Zurich, 4058 Basel, Switzerland; david.seifert@bsse.ethz.ch; 2 SIB Swiss Institute of Bioinformatics, 4058 Basel, Switzerland; 3 Division of Infectious Diseases and Hospital Epidemiology, University Hospital Zurich, University of Zurich, 8091 Zurich, Switzerland; beda.joos@bluewin.ch (B.J.); dominique.braun@usz.ch (D.L.B.); corinna_oberle@hotmail.com (C.S.O.); corinneschenkel@hotmail.com (C.D.S.); herbert.kuster@usz.ch (H.K.); christina.grube@usz.ch (C.G.); 4 Institute of Medical Virology, University of Zurich, 8091 Zurich, Switzerland; boeni.juerg@virology.uzh.ch; 5 Laboratory of Virology, University Hospital Geneva, 1205 Geneva, Switzerland; sabine.yerly@hcuge.ch; 6 University Hospital Lausanne, Service of Immunology and Allergy, University Hospital Center, 1011 Lausanne, Switzerland; vincent.aubert@chuv.ch; 7 Molecular Virology, Department of Biomedicine-Petersplatz, University of Basel, 4009 Basel, Switzerland; thomas.klimkait@unibas.ch

**Keywords:** HIV-1, transmission, transmitter–recipient pairs, Selection Test in Transmission (SeTesT), Vpu, ZPHI, SHCS

## Abstract

Little is known about whether and how variation in the HIV-1 genome affects its transmissibility. Assessing which genomic features of HIV-1 are under positive or negative selection during transmission is challenging, because very few virus particles are typically transmitted, and random genetic drift can dilute genetic signals in the recipient virus population. We analyzed 30 transmitter–recipient pairs from the Zurich Primary HIV Infection Study and the Swiss HIV Cohort Study using near full-length HIV-1 genomes. We developed a new statistical test to detect selection during transmission, called Selection Test in Transmission (SeTesT), based on comparing the transmitter and recipient virus population and accounting for the transmission bottleneck. We performed extensive simulations and found that sensitivity of detecting selection during transmission is limited by the strong population bottleneck of few transmitted virions. When pooling individual test results across patients, we found two candidate HIV-1 genomic features for affecting transmission, namely amino acid positions 3 and 18 of Vpu, which were significant before but not after correction for multiple testing. In summary, SeTesT provides a general framework for detecting selection based on genomic sequencing data of transmitted viruses. Our study shows that a higher number of transmitter–recipient pairs is required to improve sensitivity of detecting selection.

## 1. Introduction

While major achievements have been made in containing the HIV/AIDS pandemic, the worldwide prevalence of people living with human immunodeficiency virus type 1 (HIV-1) is continuing to increase [1]. HIV-1 is transmitted via body fluids containing blood, semen, vaginal secretions, or breast milk. The main route of transmission occurs via sexual intercourse. On a global scale, 80% of transmissions are due to heterosexual transmission, with over 90% occurring in the resource-limited world [2].

The human body presents multiple barriers during and after transmission that HI virions have to overcome to establish a persistent infection [3]. Crossing the genital and rectal mucosa presents itself as the first physical barrier. Once in the body, HIV-1 has to evade innate and adaptive immune responses and enter a target cell in order to replicate [3,4].

All of the aforementioned hurdles have the potential to exert selective pressure on the HIV-1 genome during the establishment of the infection. Whether selection acts on the HIV-1 genome during transmission is poorly understood [5]. It depends on host factors and on the intrinsic fitness landscape of the virus itself [6]. The viral fitness landscape is an association of a positive value, the replicative fitness of the virus, to each viral genotype. Furthermore, the fitness landscape may also depend on the host environment, and can therefore also change over time [7]. It has been shown that intra-host fitness landscapes can be inferred from either cross-sectional [8] or time-series [9] data. In contrast to the HIV-1 fitness landscape during replication in a host, much less is known about the fitness landscape at transmission to a new host, i.e., the transmissibility landscape of HIV-1. This high-dimensional evolutionary parameter can only be studied in transmission events, which are much more difficult to sample compared to longitudinal samples of one patient. A better understanding of the transmissibility landscape is crucial, for instance, for designing broadly neutralizing antibodies in the quest for an HIV vaccine [10]. Broadly neutralizing antibodies work by attacking epitopes under negative selection, that is, antigenic determinants that are conserved and that need to traverse a deep fitness valley in order to escape the immune system.

Historically, finding loci of the HIV-1 genome under selection during transmission has been already approached in the mid 1990s by analyzing the HIV-1 genetic makeup in sexual transmission pairs and in mother-to-child transmissions [11,12]. Later, with the discovery of HIV-1 co-receptors, it has been observed that viral strains preferring the CCR5 co-receptor are selected for during transmission, which appears to highlight a significant difference between the transmission and the replication fitness landscape [4]. Studies on intra-host diversification indicate an incomplete switch of co-receptor usage, suggesting higher intra-host fitness for HIV-1 populations employing a mix of both CCR5 and CXCR4 as co-receptors. Other studies have shown that shorter segments of the variable regions of the envelope protein gp120 and less potential N-linked glycosylation sites are preferred during transmission and may hence confer a selective advantage particularly in transmissions of HIV-1 subtypes A, C, and D and possibly also subtype B [13,14,15]. Studying heterosexual transmission of HIV-1 has shown that on a cohort level, amino acids that are abundant in the population of HIV-infected individuals are selected for during transmission [16]. This observation supports the notion that there are unique mechanisms of selection during transmission, which differ from the selective forces acting during the arms race between the virus and the host immune response as the untreated disease progresses to AIDS.

A great deal of effort has been invested into determining the size of the transmission bottleneck, that is, the number of transmitted viruses causing a clinically persistent infection. This number cannot be observed directly and is difficult to estimate. Different studies have supported a very small number of viruses—in most cases just one—that establish infection [17,18,19,20]. This is in line with statistical expectations under a neutral transmission model, given that the likelihood of transmitting HIV-1 is very low in general [2]. Studying selection during sexual transmission events is complicated by several factors. The correct pairing of transmitter–recipient partners in sexual transmission events has been hampered greatly due to incomplete sampling, especially of the transmitting partners, as these are often unknown to the recipient. The issue is exacerbated by legal and social complications that arise from admitting transmission of an incurable disease [21].

With the advent of next-generation sequencing (NGS), viral populations can be studied in great detail, where previous single-genome amplification assays were extremely laborious and could not sample the viral population to the required depth [22]. With sufficient depth of coverage even minor variants can be detected reliably [23].

Probabilistic models of the transmission bottleneck have been formulated that account for selection in the presence of strong selection. Bergstrom et al. [24] formulated the transmission problem using the quasispecies model. Sobel Leonard et al. [25] devised a model that estimates the bottleneck size without estimating selective pressures during the early stage of establishing the disease. Lumby et al. [26] proposed a fully probabilistic model that infers selection and the transmission bottleneck. More recent models have combined intra- and inter-host selection into an overall model for global influenza evolution [27].

Here, we develop a new statistical test that accounts for the strong bottleneck the virus population experiences during HIV-1 transmission. The test takes viral genotype counts derived from patient transmitter and recipients as input to detect selection. We found that in practice detecting selection requires either a dominant variant in the transmitter disappearing in the recipient or a minor variant in the transmitter reaching fixation in the recipient after transmission. In order to test for selection, we analyzed in 30 resolved transmitter–recipient pairs all amino acid loci of the HIV-1 genome for which more than one amino acid was found in at least six transmitters. We further assessed the amino acid sequence of the V1/V2, V3, V4 and V5 loops of the gp120 locus for signatures of selection.

## 2. Materials and Methods

### 2.1. Study Design

Transmission pairs were obtained from the Swiss HIV Cohort Study (SHCS) and in the Zurich Primary HIV Infection Study (ZPHI). The SHCS is a nationwide, clinic-based cohort enrolling at least 53% of all HIV-1-infected adults ever diagnosed in Switzerland [28,29]. Approximately 80% of those patients were included in genotypic HIV-1 drug resistance testing since 1996 [28,29]. The ZPHI is an observational, non-randomized, single-center cohort enrolling patients diagnosed with an acute or recent primary HIV-1 infection (www.clinicaltrials.gov accessed on 9 December 2021; ID NCT00537966) [20,30,31]. Blood collections and sampling of plasma are scheduled every 3 and 6 months in the ZPHI and SHCS, respectively. The SHCS and the ZPHI are approved by the ethics committee of the participating institutions (Kantonale Ethikkommission Bern (#21/88), Kanton St. Gallen Ethikkommission (#12/003), Comite departemental d’éthique des specialites medicales et de medecine communautaire et de premier recours, Geneva (#01-142), Kantonale Ethik-Kommission Zürich (#EK-793), Comitato etico cantonale, Bellinzona (#Rif. CE 813), Commission cantonale d’éthique de al recherche sur l’étre humain, Lausanne (#131/01), Ethikkommission beider Basel (#688)) and written informed consent was obtained from all participants. Patients harboring HIV-1 subtypes other than subtype B were excluded in all further analyses.

### 2.2. Determining Transmitter–Recipient Relationships

**Figure 1:**
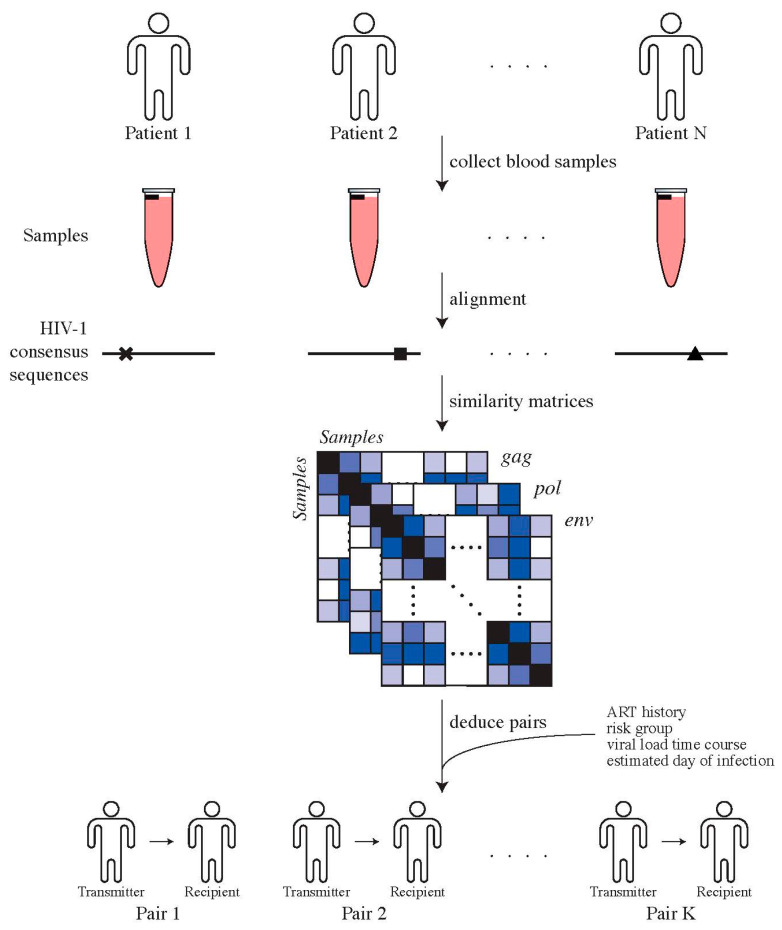
Determining transmitter–recipient relationships in the ZPHI and SHCS cohorts. We determine direct transmitter–recipient relationships by calling HIV-1 consensus sequences for all patients’ samples, generated using ngshmmalign. Pairwise similarity distances between patients’ samples were computed and combined with additional clinical and epidemiological data in order to identify transmitter–recipient pairs.

Genotypic HIV-1 drug resistance testing, i.e., protease and reverse transcriptase sequences, were used to identify transmission pairs by assessing phylogenetic relationships, as previously described [31]. To confirm potential transmission pairs, near full-length HIV-1 genome sequences, clinical and laboratory data, for instance, viral loads and transmission risk group, were used. For the potential recipients, we used the first available plasma sample for sequencing of near full-length HIV-1 genomes. For potential transmitters, we chose the closest plasma sample to the estimated date of transmission (EDT), which could also be prior to transmission (Figure 1). This procedure resulted in 30 high-confidence transmitter–recipient pairs (Supplementary Materials, Section S1).

The estimated date of transmission was determined as previously described [30]. Briefly, clinical and laboratory data such as known risk situations, appearance of first symptoms, earlier negative HIV-1 test results, avidity assays and Western blot results were considered in the estimation. Infections within 90 and 180 days after the EDT were defined as acute and recent, respectively [30].

### 2.3. Next-Generation Sequencing (NGS) Data Generation and Analysis

Viral genomic sequences were generated via the Illumina MiSeq Desktop Sequencer v2 2 × 250 kit for primary virus isolate and plasma full-length sequencing as described in [32]. Sequencing was performed using five amplicons covering the HIV-1 genome from the 5′ LTR to the 3′ LTR. In the first step of our analysis, we merged all Illumina MiSeq runs per patient. We performed clipping and quality filtering using PRINSEQ [33], where we clipped bases with a Phred score below 30 in a sliding window of 10 bases from the 5′ and 3′ ends of every read. After clipping, we required reads to be at least 200 nt in length, allowed for a maximum of 4 ambiguous ‘N’ bases and required an average Phred score of at least 30 over the whole read. Reads missing a mate, either due to missing it right from the start or due to previously performed quality checks, were removed from the preprocessed data.

We aligned the quality-trimmed paired-end reads using the custom-made aligner ngshmmalign (https://github.com/cbg-ethz/ngshmmalign accessed on 9 December 2021) [23]. Briefly, this alignment tool employs a profile Hidden Markov Model (HMM) to account for insertions and deletions that occur as part of the natural evolution of HIV-1. Certain loci, such as p6, gp120 and nef are prone to structural variants. The hypervariable V1 to V5 loops in gp120 are especially likely to include a large number of insertions and deletions relative to the reference strain HIV-1 HXB2 (GenBank accession number K03455). ngshmmalign accounts for biological insertions and removes conserved deletions with respect to the cohort consensus sequence to build a new consensus sequence. This patient-specific consensus sequence contains all insertions with respect to each patient, such that deletions make up all real structural variants per patient.

In order to standardize all patients to the same reference genome, we performed a multiple sequencing alignment of all patient consensus sequences and HIV-1 HXB2 using MAFFT [34]. The resulting multiple sequence alignment was used to convert each patient’s alignment to a standardized alignment against HIV-1 HXB2, resulting in comparable loci between patients with respect to a standardized HIV-1 reference. From these alignments, we extracted the amino acid composition at every residue site of all open reading frames. We retained only amino acids with frequency above 0.75% at every locus, in order to avoid calling spurious variants arising due to sequencing or RT-PCR errors. The cutoff 0.75% represents a tradeoff between losing minor variants and calling variants erroneously (false positives). In all but the most extreme datasets analyzed by Schirmer et al. [35], 0.75% is higher than the mean error rate across both mate pairs.

After running our analysis pipeline, we have genomic consensus sequences of majority nucleotide bases, genomic consensus sequences with ambiguous nucleotide bases of more than 5% minor allele frequency, amino acid allele frequencies at every position of every open reading frame above 0.75% minor allele frequency and amino acid haplotypes in the hypervariable regions of gp120. Details of the next-generation sequencing characteristics are given in Supplementary Materials, Section S2.

### 2.4. Testing for Selection during Transmission

**Figure 2:**
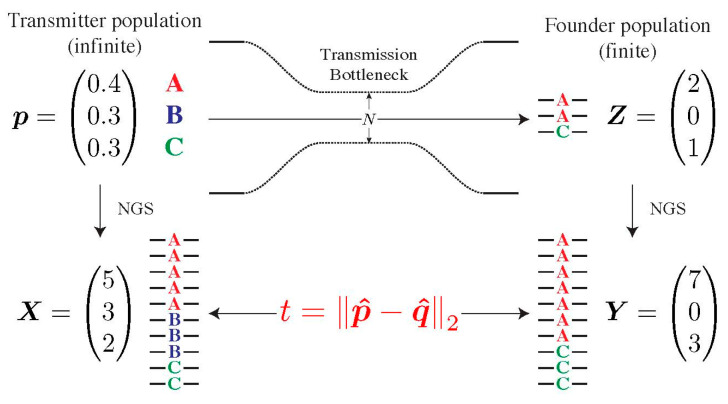
Modelling the transmission bottleneck. The transmission bottleneck of the HIV-1 population shapes its composition in the recipient drastically. Starting with an infinite pool of three genotypes A, B and C in the transmitter ***p***, our model estimates deviations from neutrality by first passing this population through a strong bottleneck, yielding a small founder population in the recipient ***Z***. Both the transmitter and founder population cannot be estimated directly, and can only be sampled by NGS, yielding the vector of counts ***X*** and ***Y***. The Euclidean distance t between vectors ***p*** and ***q*** of relative abundances defines our test statistic.

To determine whether the composition of the transmitted population in the recipient deviates from the one of the presumed transmitters, we developed a statistical test, which we coined Selection Test in Transmission (SeTesT, https://github.com/cbg-ethz/SeTesT accessed on 9 December 2021); a detailed description can be found in Supplementary Materials, Section S3). The genotypes we test for can be single-locus amino acids, nucleotides, or haplotype blocks, i.e., sequences of nucleotides or amino acids spanning multiple loci. The input data is a 2 × *K* contingency table of absolute counts, where *K* denotes the number of genotypes. Entries in the two rows denote the number of times a genotype occurs among all sequencing reads obtained from the transmitter and recipient, respectively (Figure 2).

As the transmitted population undergoes a strong population bottleneck due to physical and immunological barriers, general methods for 2 × *K* contingency tables cannot be employed, as they only capture the sampling variance and fail to account for other sources of variation, most notably downsampling during the transmission bottleneck. Therefore, in practice, such tests suffer from massively inflated false positive rates (Supplementary Materials, Section S4).

To address this limitation, we developed SeTesT, a bespoke statistical test that accounts for variation in the unknown size of the transmission bottleneck (Supplementary Materials, Section S3). Briefly, we developed a probabilistic graphical model to derive the genotype counts of both the transmitter and recipient virus population from the viral NGS data (Supplementary Materials, Section S3.1). We tested the null hypothesis that the fitness landscape during transmission is flat, that is, all genotypes have the same fitness (Supplementary Materials, Section S3.2). The alternative hypothesis is that there exists at least one genotype that possesses a selective advantage during transmission. Additionally, we modelled the bottleneck explicitly using a single-generation Wright-Fisher process, where we assumed a mean bottleneck size of around 1.78 virions per transmission event [17] (Supplementary Materials, Section S3.3). SeTesT test is based on comparing the genotype distributions directly using the Euclidean distance, rather than estimating any model parameters. Significant differences between the two distributions indicate deviation from neutrality during transmission. For multi-locus genotypes, we adapted the test statistic to account for divergent evolution between transmitter and recipient genotypes during the time from transmission to sample collection (Supplementary Materials, Section S3.4).

### 2.5. Assessing the Specificity of Our Model

A crucial part for every statistical test is ensuring that it is statistically correct, that is, the probability of yielding a *p*-value smaller than α is at most α. Such tests are also referred to as unbiased [36]. Tests that yield on average a higher fraction of *p*-values below α for some given α are considered liberal or anti-conservative and do not allow for controlling the false positive rate. We simulated data from our model for different population settings and for different read coverages in the transmitter and analyzed whether our test yields inflated *p*-values or not (Supplementary Materials, Section S4).

### 2.6. Assessing the Sensitivity of SeTesT

In order to detect selection, we tested the statistical power, or sensitivity, of SeTesT by simulating a multinomial sampling process, followed by a read simulation step, and then running our test on these simulated data (Supplementary Materials, Section S5). We tested the sensitivity over a range of frequencies and fitness values of a single genotype in the population having a positive or negative selective advantage. In addition, we assessed sensitivity for different read coverages in the recipient. Finally, we assessed sensitivity given 2, 3, 5, and 10 different genotypes in the transmitter.

### 2.7. Combining Data Sets to Increase Power

As different pairs of transmitters and recipients can be regarded as independent, multiple pairs can be combined into one summary statistic in order to gain power. We employed Fisher’s method to combine multiple *p*-values of different transmitter–recipient pairs for the same locus. Fisher’s sum-of-logs test has been shown to be one of the most powerful tests for combining independent tests in the setting of genome-wide association studies [37]. In order to assess the gain of power through pooling of pairs of patients, we simulated from our model and assessed the sensitivity of our model when using differing numbers of pooled *p*-values.

### 2.8. Assessing the Effect of Early Sampling

In order to estimate the effect of an evolving viral population on the results of our test, we have selected transmitter–recipient pairs 1, 21, and 29 (Supplementary Materials, Section S2), which all have less than 30 days to the estimated date of infection. By focusing on just these pairs, we can exclude selection affecting the population and changes in genotype distribution can more reliably be attributed to transmission alone.

## 3. Results

We developed a statistical test called Selection Test in Transmission (SeTesT) on the basis of a probabilistic model for detecting selection during viral transmission using viral NGS data of transmitter–recipient pairs (Figure 2). The test accounts for the strong population bottleneck during transmission, the finite sampling of the underlying virus populations that NGS data provides, and divergence of multi-locus genotypes due to the time-span between transmission and sampling. Simulating from the probabilistic model, we confirmed that SeTesT is capable of controlling the false positive rate. Below we first assess the statistical sensitivity of SeTesT under various parameters and then analyze NGS data from 30 curated transmitter–recipient pairs to detect selection during transmission in the HIV-1 genome.

### 3.1. Detecting Selection Is Compromised by the Transmission Bottleneck

We performed simulations with different transmission fitness landscapes in order to assess the power of our test to detect selection during transmission. We found that a significant obstacle in detecting transmission selection is the strong population bottleneck (Supplementary Materials, Section S4), while read coverage of the transmitted viral population does not improve statistical power beyond 100–1000 reads. SeTesT is very conservative for a regime in which only 1–2 viruses are likely transmitted (Supplementary Materials, Section S4.1). We found *p*-values to be inflated by a factor of 17.4 (Supplementary Materials, Section S4.1). In general, with an increasing number of genotypes, the test becomes less conservative (Supplementary Materials, Section S4.3).

The statistical power of SeTesT strongly depends on the composition of the population at the time of transmission (Supplementary Materials, Section S5), but it is practically independent of the read coverage of the recipient viral population (Supplementary Materials, Section S5.1). Detecting selection at a single locus is only possible when this specific genotype (i.e., nucleotide or amino acid) with high selective advantage exists at a low frequency in the transmitter and then reaches a very high frequency, which typically means fixation, in the recipient after transmission. On the other hand, detecting selection against a single genotype requires the other genotypes that are selected for to be minor variants in the transmitter. Detecting selection against a multi-locus genotype becomes more and more feasible for an increasing number of loci (Supplementary Materials, Section S5.2), as the increasing number of possible genotypes makes the test less conservative. At the same time, detecting selection for a single locus is not aided by an increasing number of observed genotypes in the population, because only a situation in which the selected genotype reaches fixation can lead to a significant result.

**Figure 3:**
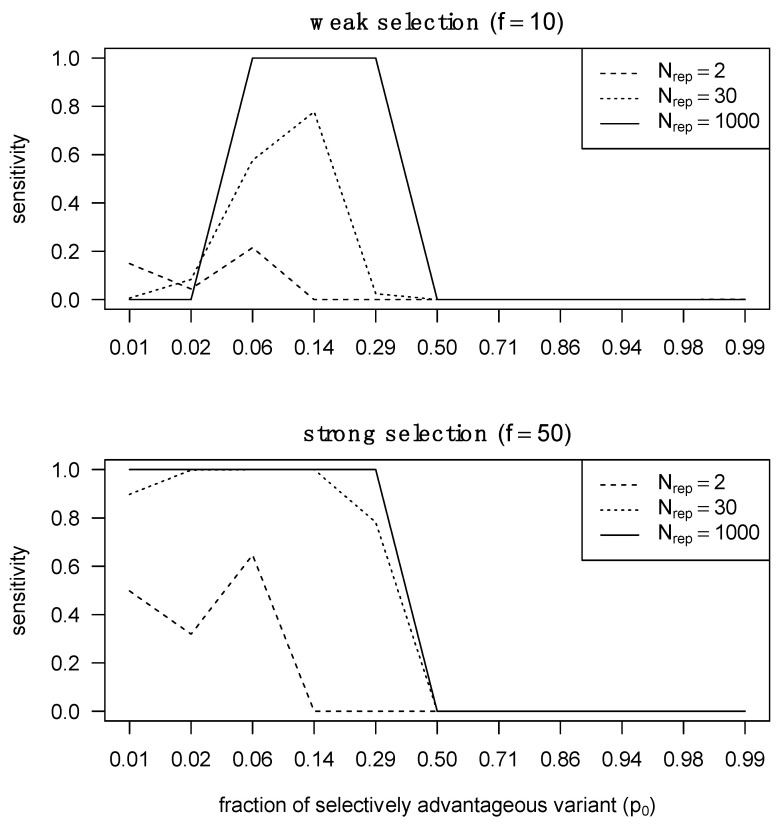
Sensitivity of detecting selection in different selection regimes with different numbers of pooled transmitter–recipient pairs. We assessed statistical sensitivity of detecting selection across 2, 30 and 1000 pairs. On the right, we performed the same analysis but in the presence of strong selection.

Aggregating *p*-values for the same locus across multiple patients allows for near-perfect sensitivity with more than 30 transmitter–recipient pairs when either a genotype selected for in the recipient had a low frequency in the transmitter, or a highly frequent transmitter genotype was selected against and in the recipient. On the other hand, when a selected genotype is already abundant in the transmitter population or a genotype being selected against is absent from the transmitter population, little sensitivity can be achieved, even with a large aggregation of *p*-values across patients (Supplementary Materials, Section S5.3). Thus, we can detect selection during transmission if we observe an aggregate of minor variants being the source of the founder population. Detecting weak selection is significantly more challenging than detecting strong selection, because the required proportion of the selectively advantageous variant must pre-exist in the transmitter at a certain fraction that is neither too low nor too high (Figure 3). At extremely low abundances, the selectively advantageous variant cannot rich fixation with only weak selection, whereas under strong selection the selected variant can reach fixation over a larger range of relative abundances.

### 3.2. Assessing Selection in the HIV-1 Genomes of ZPHI and SHCS Transmitter–Recipient Pairs

We proceeded to determine likely transmitter–recipient pairs from previous exploratory studies on the ZPHI/SHCS cohorts based on the whole HIV-1 genome (Figure 2). We matched recipients to their prospective transmitters by determining empirical pairwise distances and acceptable cut-off thresholds on the overall distribution of these distances. Combined with ART history, risk group, viral load time course, and estimated date of infection we determined the final transmitter–recipient relationships (Supplementary Materials, Section S1). In total, we determined 30 high-confidence transmitter–recipient pairs. As part of this analysis, we could recover seven out of eight confirmed transmitter–recipient pairs that have been determined and comprehensively validated in another study on the basis of multiple assays on the env locus [31]. The most common route of transmission was between MSM (men who have sex with men), 28/30 (93%). The majority of transmitters were in the chronic phase of their HIV-1 infection at the estimated date of infection, 21/39 (70%). The median sampling date was 53 (IQR: −4; 96) and 49 (IQR: 31; 77) days after the estimated day of infection for transmitters and recipients, respectively (Supplementary Materials, Section S1).

**Figure 4:**
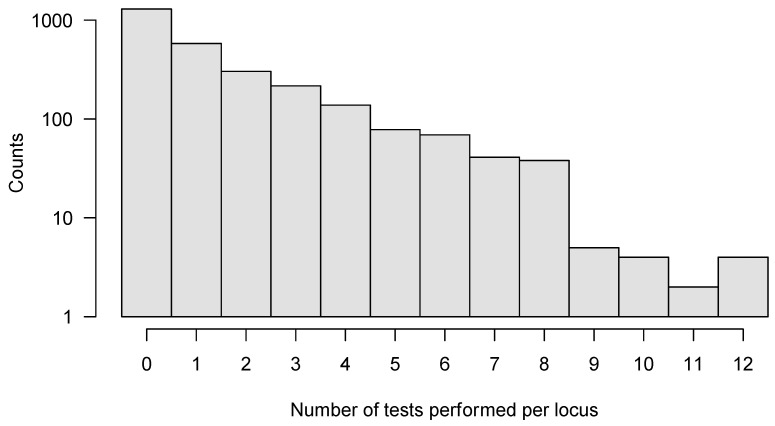
Histogram of number of tests per locus that could be performed for the whole HIV-1 genome. In total, we analyzed 2773 individual amino acid loci, with a maximum of 12 tests performed for any locus. In order to improve sensitivity, we only analyzed those loci further where six or more tests could be performed out of all 30 pairs.

We applied SeTesT in a whole-genome fashion to each amino acid at each single locus of the HIV-1 genome, excluding the heterogeneous V1/V2, V3, V4 and V5 loops of the gp120 open reading frame, because these hypervariable regions showed a very high degree of heterogeneity in multiple sequence alignments [38,39] leading to spurious calls (Supplementary Materials, Section S2). Instead, we considered multi-locus genotypes in these regions and analyzed the complete protein sequences of the V1/V2, V3, V4 and V5 loops of gp120 open reading frame. We analyzed a total of 2773 individual amino acid loci of the HIV-1 genome (Supplementary Materials, Section S6). Approximately half of the loci (1296) could not be tested, due to lack of multiple amino acids in the transmitter or failed amplicons. A further 579 loci allowed only for one test across the 30 pairs. We could perform a maximum of 12 tests for four loci (Figure 4). Assessing the impact of early HIV-1 infection (sampled within 30 days) compared to established HIV-1 infection (over 30 days) yielded no significant locus under selection after pooling across pairs.

**Figure 5:**
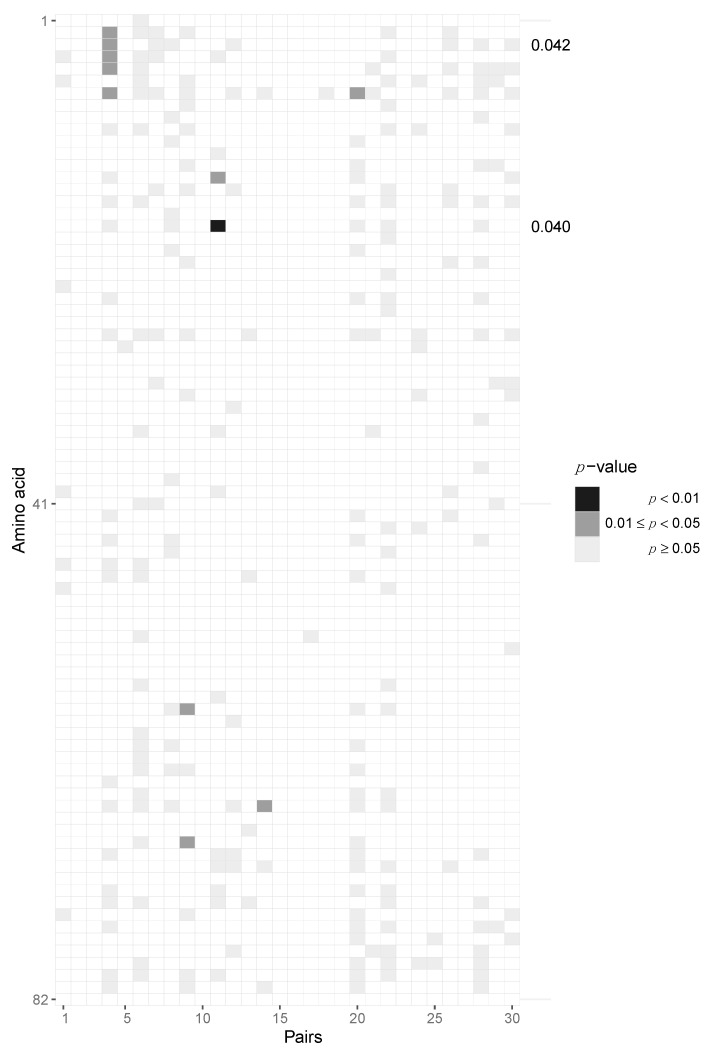
Potential selection of Vpu variants during HIV-1 transmission. Heatmap of *p*-values of the Vpu protein. Each column represents the test outcomes for a transmitter–recipient pair and every row represents one amino acid locus out of 82 across all 30 recipient and transmitters. Significant *p*-values after pooling across pairs without Benjamini–Hochberg multiple testing correction are shown on the right axis. Amino acid loci 3 and 18 were found to be significant.

**Figure 6:**
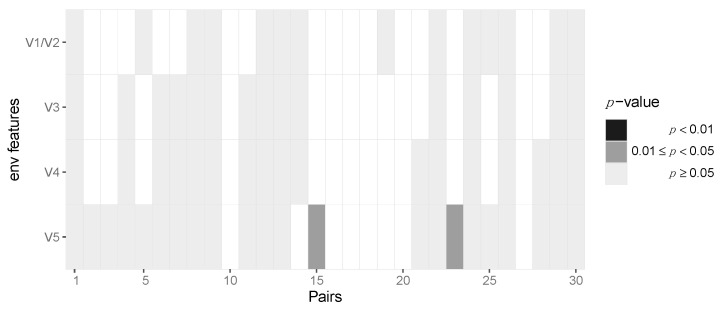
No evidence for selection of Env gp120 V region variants during HIV-1 transmission. Heatmap of *p*-values of the gp120 immunogenic loci. Each column represents the test outcomes for a transmitter–recipient pair. The *p*-values across the 30 transmitter–recipient pairs for the four immunogenic loci (V1/V2, V3, V4 and V5) are shown on the x and y axis, respectively.

After aggregating *p*-values by pooling across patients for each locus, we required at least six pairs out of 30 to have at least two amino acids in the transmitter and one in the recipient in order to be able to perform the test, resulting in 163 potential sites across protein-coding regions of the HIV-1 genome. After pooling the data in this fashion, we identified two amino acid loci, namely positions 3 and 18, of Vpu that are statistically significant before multiple testing correction (*p* = 0.042 and 0.04, respectively) but not after Benjamini–Hochberg FDR correction (Figure 5, Supplementary Materials, Section S6). At amino acid position 3, we found serine-to-alanine and proline-to-serine transitions being potentially selected for during transmission. At residue 18, we found the leucine-to-isoleucine transition to be selected for. None of the immunogenic loops in gp120 showed any signatures of selection when pooled across all pairs of patients (Figure 6).

## 4. Discussion

We have developed SeTesT, a customized deep sequencing-based statistical test for deviations from a neutral transmission model taking into account the population bottleneck of viral transmission and potential divergence of transmitted multi-locus genotypes. We considered the entire HIV-1 genome in 30 curated transmitter–recipient pairs and tested those loci for which at least six tests could be performed across pairs. We found two loci under selection before multiple testing correction.

With our whole-genome analysis, we found parts of Vpu to be possible candidates for being under selection. Given that at amino acid position 3 no single amino acid was selected for, the locus may be under positive selection. This is also corroborated by the high inherent heterogeneity of Vpu, which is more in line with positive selection than purifying negative selection. Vpu has been shown to affect virion release in transmitter–recipient pairs [40,41]. Both loci are in the transmembrane part of Vpu, which are determinants of CD4 downregulation [42].

SeTesT models the transmission event by sampling from the transmitter’s viral population a very small number of virions that are potentially passed on to the recipient. We model this stochastic process to derive a test statistic over the observed data. We assume that the estimates of the viral variants derived from the read data are unbiased and drawn from a multinomial distribution. In case the NGS population frequency estimators are known or believed to be biased with respect to the true frequencies, the primerID protocol allows for certain error corrections [43,44].

We have shown that SeTesT produces meaningful *p*-values and in particular controls the type I error rate. It is, however, very conservative when assuming that on average 1.78 viruses are transmitted per event for successful transmission events. The power of the test is effectively determined by this bottleneck, with all other parameters having much smaller effects on sensitivity. Ignoring this bottleneck would lead to a massively inflated false positive rate, as can be seen, for instance, when just using Fisher’s exact test. This effect seems intuitive when regarding the number of transmitted viruses as independent replicates of an experiment. Having just one observation, i.e., one transmitted virus, will not be enough evidence to falsify the null hypothesis of neutral transmission and stochastic sampling alone. A proper experimental way to increase power would be to repeat the transmission with the same transmitter population, conditioned on knowing the true transmission network. Such a repeated transmission under controlled conditions is, however, for ethical, experimental, biological and practical reasons impossible in humans. An in vitro model, where the different genetic bottlenecks are separated, could be a viable validation strategy of candidate loci under selection. With a two compartment in vitro model, where the two compartments are separated by an artificial mucosa [45], characteristics of the virus may be tested that make it more permeable to the mucosal layer. In a second in vitro system one might utilize a well-characterized culture of cells of the human innate immune system, in which the transmissibility with respect to the immune system could be tested.

The number of transmitted virions in our model corresponds to the known infection risk per intercourse for men who have sex with men [46]. With approximately 1.78 viruses transmitted during such a transmission event, under a Poisson model, the estimated probability of transmission is approximately 40%. This probability is much larger than any known figure from epidemiological studies [47], suggesting that the transmission bottleneck may be even smaller than 1.78 viruses in practice when not conditioning on successful transmission. In this sense, our bottleneck parameter can be considered to be on the liberal side. We further make the implicit assumption that the population frequencies at the sampling time are representative of the frequencies during transmission. To what extent this assumption is violated is hard to judge, given that we have no longitudinal data. To our knowledge, there are no comprehensive studies that have analyzed the stability of variant frequencies from time of infection to when blood is sampled.

We have introduced an amino acid substitution model for cases where whole open reading frames are to be tested for selection and recipient sequences are not a subset of transmitter sequences. In general, substitution models assume that all sites in a sequence are not under any selective pressure. While we cannot ascertain neutrality for all sites, even in cases of non-neutral transmission we use the substitution model only to match recipient sequences to transmitter sequences and not for any phylogenetic inference or the test itself. Furthermore, the sampling times are two orders of magnitude lower than the reciprocal of the substitution rate, such that mismatching transmitters to recipients is very unlikely.

It should be noted here that we have analyzed our test in a fashion similar to genome-wide association studies (GWAS). Importantly, pooling across patients is statistically valid, as different pairs of transmitter and recipients are independent of each other given the transmission network. Such pooling methods are common practice in the field of GWAS [37]. Because most likely only one virus is transmitted in the majority of transmissions, linkage disequilibrium is maximal, as initially the newly established infection cannot reduce linkage equilibrium by recombination for a lack of other haplotypes. With this strong linkage disequilibrium, *p*-values along the genome are not statistically independent, making our test on a whole-genome scale substantially more conservative than if sites were independent. Our analysis is in contrast to other analyses on the level of cohort features, such as those performed in [16]. Our goal is to detect selection on individual amino acids and the different immunogenic loops of gp120, whereas the authors in [16] have focused on identifying summary statistics that are supported on a population level, without determining individual loci under selection. Furthermore, their study has excluded the env locus, whereas we include all of gp120 and all of gp41 in our analysis.

While the final result involves 163 sites on which multiple testing correction was applied, the effective number of tests is much lower, that is, the hypothetical number of independent tests if all correlation due to linkage disequilibrium between tests is taken into account. Furthermore, due to the conservative nature of our test, most *p*-values are larger than statistically necessary due to strong discrete effects in the test statistic [48]. For these reasons, the two sites in Vpu may still be regarded as interesting candidates for selection despite not reaching statistical significance after correction for multiple testing. Further investigations are required to falsify or confirm their involvement in non-neutral transmission.

Another study has also concluded that minor variants are preferentially transmitted, corroborating that selection acts during transmission, without being able to pinpoint any one locus that appears to be under selection [49]. The authors found that the transmitted variant is not the majority variant of the transmitter’s genital tract. A limitation of our method is that we cannot detect complex higher-order patterns of selection, where the selective advantage derives not from the effect of a single allele, but the interaction of multiple alleles that are not observable within the same sequencing reads. In this scenario, the space of the test statistic space grows exponentially and we would lose all statistical power.

Another limitation of our method is that we assume that we have uncovered the true direct transmitter–recipient pairs. Phylogenetic methods on the other hand do not require explicit pairs of transmitter–recipient relationships because they estimate the transmission network implicitly. While being able to estimate evolutionary divergence and the potential indirect transmission network, separating the evolutionary pressures from transmission and intra-host evolution becomes more involved if only the former is to be investigated. Furthermore, early immune escape and reversion are potential confounding factors that are impossible to control for in our setting, because this would require longitudinal data to tease apart different evolutionary forces [50,51].

The number of patients in our study is limited but still considerable given the difficulty of reliably matching transmitters with recipients, not least due to involved legal and ethical intricacies. We have validated the pairs using multiple sources of anamnestic records, and have found that a subset of pairs in our study are in close agreement with pairs from our previous study [31]. We believe that increasing sensitivity in detecting transmission selection can only be achieved through a concerted effort of the HIV community to pool cohorts. Only when the number of properly paired transmitter–recipient samples is drastically increased can we hope to improve sensitivity of detecting selection. Our statistical analysis shows, for example, that if the transmitting populations are assumed to have the same population composition, then for 1000 aggregated patients, selection can be detected with sensitivity 1.0.

## 5. Conclusions

We have developed SeTesT, a novel statistical test to determine whether selection acts on certain parts of the HIV-1 genome during sexual transmission events. Applying the test to 30 transmitter–recipient pairs, we have found new potential candidates for selection in Vpu. Our probabilistic model also shows that statistical inference in general is very challenging and we quantify its limitations to help design future studies. Our results suggest that a higher number of transmitter–recipient pairs is required to improve sensitivity of detecting selection.

SeTesT can be used in other settings than sexual transmission events and for different viruses. For instance, it could also be used for estimating selection on the genome of the Hepatitis C virus, given that the genetic bottleneck for an intravenous transmission mode could be estimated. Thus, SeTesT provides a tool based on a statistically sound approach for exploring the role of selection during viral transmission events.

## Data Availability

The HIV sequence data was obtained as part of the Zurich Primary HIV Infection Study and of the Swiss HIV Cohort Study (SHCS), whose authors may be contacted at www.shcs.ch/contact, accessed on 9 December 2021. Due to the representativeness of the dataset, the sensitivities associated with HIV infections, and to protect the privacy of patients enrolled in this study, a deposition of all sequence data in an open database is not possible at this time.

## Supplementary Materials

The following supporting information can be downloaded at: https://www.mdpi.com/article/10.3390/v14020406/s1. The supplementary materials contain supplementary information in six sections. Section S1. Pairing recipients with potential transmitters, Section S2. Next-generation sequencing statistics, Section S3. SeTesT: A statistical test for selection during transmission, Section S4. Validating the false positive rate, Section S5. Sensitivity of detecting selection, and Section S6. Heatmaps and number of genotypes across the HIV-1 genome.
