# Supplementary material for: Detecting Selection in the HIV-1 Genome during Sexual Transmission Events
Source: Viruses. 2022 Feb 16;14(2):406. doi: 10.3390/v14020406 (PMC8876189; doi:10.3390/v14020406)
Supplement: Supplementary file 1 [file viruses-14-00406-s001.zip › viruses-1525203-supplementary.pdf]

Supplementary Information

In section S1, we demonstrate how we determined likely direct transmission events. Section S2 contains all the sample-related metrics for the inferred transmission pairs. In section S3, we introduce the mathematical model used in our test for inferring selection. We check that the model produces correct p-values in section S4 and assess the sensitivity to detect selection in section S5. Finally, section S6 lists the heatmaps for number of amino acids per position within every HIV-1 reading frame and p-values for all tests performed.

S1 Pairing recipients with potential transmitters

In order to pair transmitters, who may have transmitted the virus, with their respective recipients, we consider the similarity between the consensus sequences of the HIV-1 population they harbour and further clinical and epidemiological parameters. We first calculate a distance score between all samples of all patients in the ZPHI and SHCS cohorts and combine this data with ART history, risk group, viral load time course, and estimated day of infection to determine the final transmitter-recipient relationships. Here, we define the similarity distance ranking between the viral consensus sequences.

Due to intra-host viral genetic heterogeneity, consensus sequences can contain ambiguous characters, such as for instance `K`, which denotes a multi-allelic locus where both `G` and `T` occur. They are thus defined as strings over an extended alphabet, the IUPAC nucleotide code, ignoring indels. Let $\boldsymbol{\alpha}$ and $\boldsymbol{\beta}$ be two such aligned sequences of length L. We generalise the Hamming distance and define the non-symmetric transmitter-to-recipient distance

$$d(\boldsymbol{\alpha},\, \boldsymbol{\beta}) = 1 - \frac{1}{L}\sum_{i=1}^{L} \frac{|\alpha_i \cap \beta_i|}{|\beta_i|} \in [0,1] \tag{S1.0-I}$$

where α_i and β_i denote the expanded set of all bases at locus i in consensus sequence $\boldsymbol{\alpha}$ and $\boldsymbol{\beta}$, respectively. For example, for transmitter sequence `GGG` and recipient sequence `KKK`, we find

$$d\,(\texttt{GGG},\, \texttt{KKK}) = 1 - \frac{|\{\texttt{G}\} \cap \{\texttt{G},\texttt{T}\}|}{|\{\texttt{G},\texttt{T}\}|} = 0.5$$

whereas if we consider transmitter and recipient interchanged

$$d\,(\texttt{KKK},\, \texttt{GGG}) = 1 - \frac{|\{\texttt{G},\texttt{T}\} \cap \{\texttt{G}\}|}{|\{\texttt{G}\}|} = 0$$

In this example, the transmission `KKK` $\rightarrow$ `GGG` is more likely, because the sequences are closer to each other, than `GGG` $\rightarrow$ `KKK`. The rationale lies in not penalising heterogeneity in the transmitter due to many ambiguous bases, but rather in how well the bases of the recipient can be explained as a subset of bases of the transmitter. If both sequences contain no ambiguous characters, then (S1.0-I) reduces to the (symmetric) normalised Hamming distance.

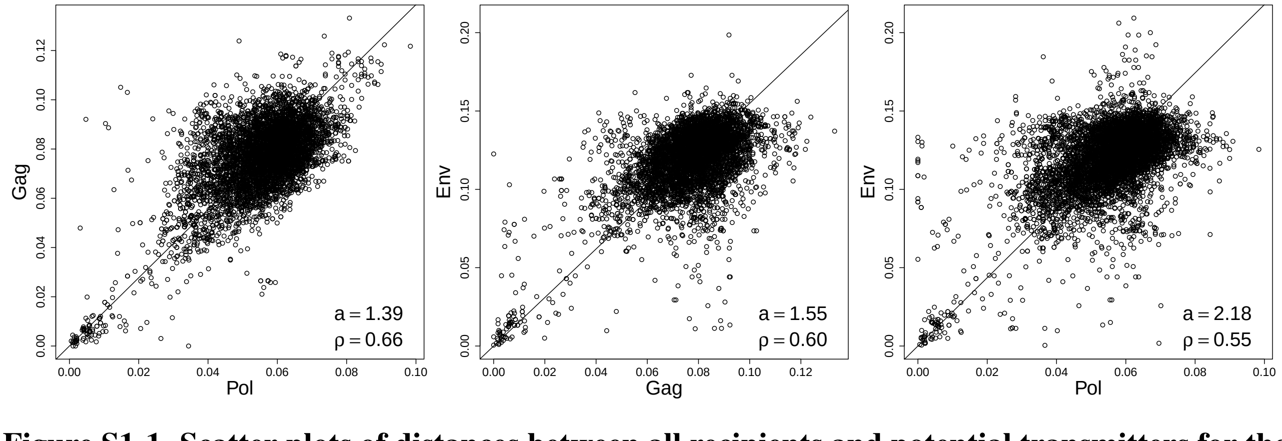

Figure S1-1. Scatter plots of distances between all recipients and potential transmitters for the three genes *gag*, *pol* and *env*. The solid line represents the fit of the Deming regression and its slope is denoted by a. The correlation between the distances of gene pairs is denoted by the Pearson correlation coefficient ρ.

In order to judge whether a distance between a recipient and a prospective transmitter can be considered significantly close, we determine empirical thresholds on the basis of the background distribution of these relative distances between unrelated recipients and transmitters. The background distribution consists of the entirety of all computed pairwise distances and is described by the sample mean μ and sample standard deviation σ. To account for heterogeneity in substitution rates, we calculate relative transmitter-to-recipient distances for the *gag*, *pol* and *env* genes separately. From the distribution of distances, we normalise the distances of *gag* and *env* to the *pol* distance by performing a Deming regression with the intercept fixed to the origin (Beijk *et al.* (2008), Figure S1-1). Finally, the average genomic distance between a recipient and potential transmitter is the mean of these three normalised distances (or any subset of them if an amplicon happened to have failed during sample preparation). The lower acceptable threshold for a transmitter-recipient pair is then $\mu - 3\sigma = 2.0\%$, and the upper acceptable threshold is $\mu - 2\sigma = 3.2\%$. Distances below the lower threshold are strong indicators of a possible transmission pair, distances between the lower and upper threshold are only considered with strong diagnostic or expert-guided anamnestic evidence, and distances above the upper acceptable threshold are not considered for a transmission relationship and will be discarded.

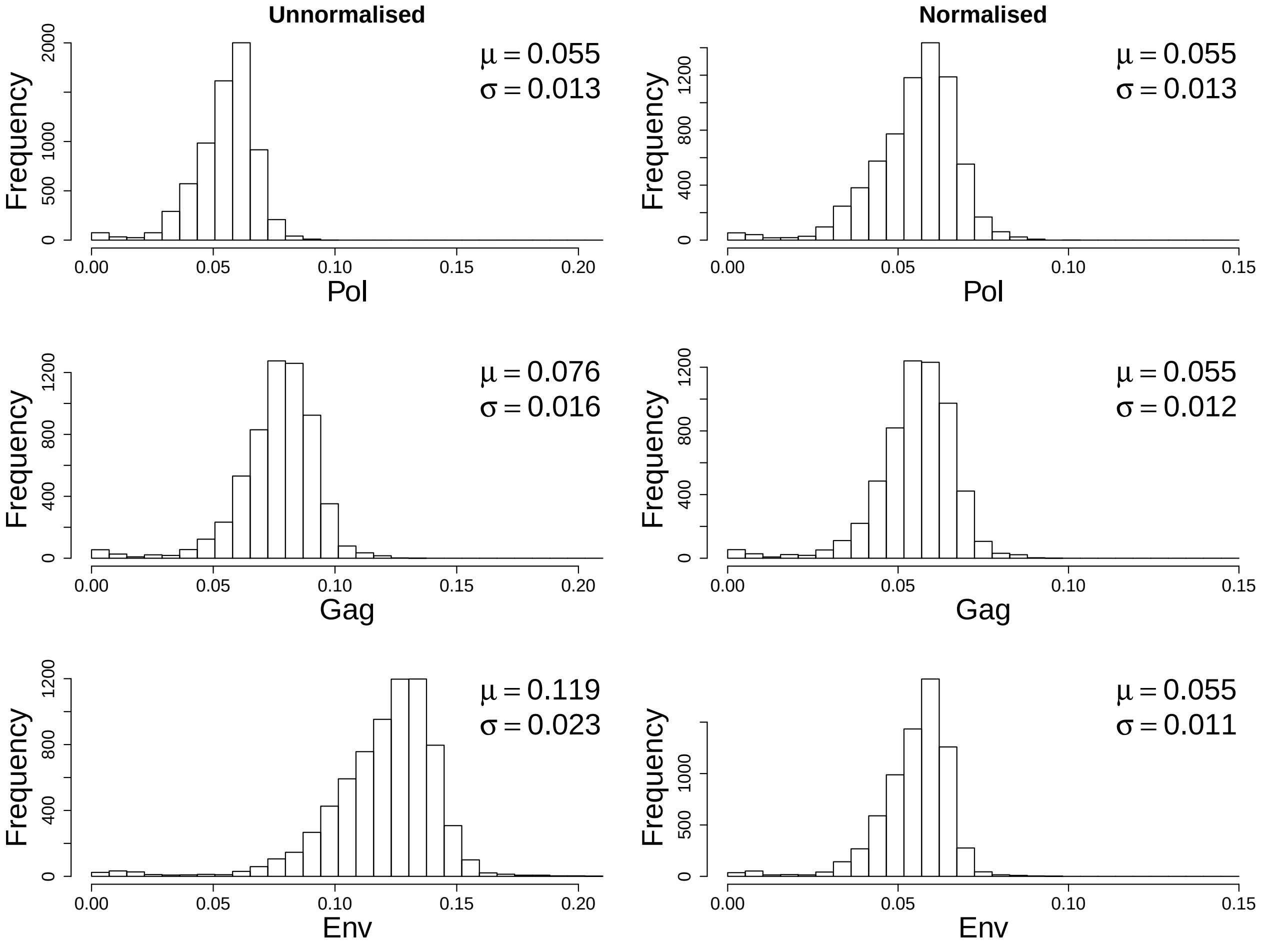

Figure S1-2. Histograms of distributions of distances between all recipients and potential transmitters for the three genes *gag*, *pol* and *env*. The histograms on the left represent unnormalised differences. The histograms on the right represent the distances normalised to the levels of *pol* by dividing their distances by the slope of the Deming regression from Figure S1-1.

This procedure resulted in a total of 30 matched transmitter-recipient pairs. They are listed in Table S1-1 with clinical co-variates and their pairwise consensus sequence distances.

Pair		Transmission mode	Interval in days	Viral load HIV-1 RNA copies / ml plasma	CD4 count cells / ml blood	Infection stage of transmitter	d_{weighted} [%]	d_{gag} [%]	d_{pol} [%]	d_{env} [%]
1	T	MSM	−20	43,200	422	chronic	0.1	0.2	0.1	0.1
	R		26	373,000	430					
2	T	MSM	−451	1,090,000	493	chronic	0.9	1.1	0.9	2.2
	R		60	1,630,000	474					
3	T	MSM	33	2830	595	chronic	0.3			0.7
	R		61	5,970,000	362					
4	T	MSM	64	204,000	619	acute	0.8	1.2	1.3	0.9
	R		63	191,000	355					
5	T	MSM	−4	967,000	427	acute	0.0	0.0	0.1	0.1
	R		35	168,000	362					
6	T	MSM	96	87,000	367	acute	0.4	0.2	0.2	1.7
	R		103	8960	325					
7	T	HET	49	18,300	416	chronic	1.6	0.3	2.6	4.5
	R		42	412,857	625					
8	T	MSM		160,500	506	chronic	0.3	0.6	0.4	0.5
	R			21,100	N/A					
9	T	MSM	−52	101,500	325	chronic	0.8	1.1	0.5	2.5
	R		81	149,000	400					
10	T	MSM	60	19,800	617	N/A	0.2	0.3	0.2	0.6
	R		31	2,220,000	643					
11	T	MSM	170	34,600	115	chronic	0.4	0.7	0.4	0.6
	R		84	14,400	656					
12	T	MSM	53	111,000	449	chronic	0.3	0.2	0.4	0.7
	R		21	105,000	327					
13	T	MSM	−146	4310	356	chronic	0.5	0.5	0.5	1.4
	R		28	1,090,000	493					
14	T	HET	63	257,532	623	recent	0.1	0.1	0.2	0.2
	R		49	428,000	N/A					
15	T	MSM	52	8780	472	chronic	0.4			0.8
	R		56	56,300	564					
16	T	MSM	−860	6470	384	chronic	1.2	1.0	1.6	
	R		491	10,200	437					
17	T	MSM	86	26,000	293	chronic	0.5	0.7	0.4	1.5
	R		29	170,000	300					
18	T	MSM	145	11,700	1024	acute	0.1	0.2	0.1	0.4
	R		117	146,000	321					
19	T	MSM	−721	583,000	N/A	chronic	1.1	1.8	1.0	1.9
	R		77	470,000	296					
20	T	MSM	123	1950	430	chronic	0.6		0.3	1.8
	R		22	50,800	603					
21	T	MSM	−7	60,200	378	chronic	0.7	2.0	0.5	0.5
	R		21	430,000	302					
22	T	MSM	229	227,071	500	chronic	0.8	0.6	1.0	1.9
	R		154	173,379	489					
23	T	MSM	69	38,109	196	acute	0.2	0.4	0.1	0.5
	R		49	10,000,000	287					
24	T	MSM	270	133,000	509	chronic	0.6	0.6	0.6	1.4
	R		66	97,000	174					
25	T	MSM	52	23,800	506	N/A	0.3	0.2	0.2	0.8
	R		55	2,610,000	315					
26	T	MSM	335	691,830	380	chronic	0.2	0.4	0.2	0.4
	R		42	3560	559					
27	T	MSM	61	24,042	325	chronic	0.4	0.2	0.5	1.4
	R		110	40,763	468					
28	T	MSM	237	208,000	197	chronic	0.4	0.2	0.8	0.8
	R		35	29,300,000	226					
29	T	MSM	2	94,700	492	N/A	0.4	0.4	0.6	0.6
	R		30	32,800	834					
30	T	MSM	50	9440	508	chronic	0.1	0.1	0.1	0.5
	R		44	1,170,000	247					

Table S1-1. Characteristics of the 30 transmitter-recipient pairs. Here MSM denotes transmissions between men who have sex with men, whereas HET denotes heterosexual transmission. The interval in days is the time span between sample date and estimated date of infection (EDI), where negative intervals come from transmitters that were sampled before the EDI. The viral load and CD4 count have been measured at the time the sample was taken. The $d_{(\cdot)}$ columns are for the previously calculated genes respectively weighted relative distances of all genes between transmitter and recipient.

S2 Next-generation sequencing statistics

Pair		raw reads	aligned reads	fraction	amplicon A *gag – pol*	amplicon B *pol*	amplicon C *pol – vpu*	amplicon D *vpr – gp41*	amplicon E *gp120 – nef*
1	T	1,010,840	616,858	61.0%	14,262	14,965	23,677	17,078	14,805
	R	548,394	345,984	63.1%	6237	8890	6701	10,335	10,299
2	T	2,363,774	1,373,208	58.1%	24,444	33,059	52,330	32,168	23,583
	R	383,880	283,802	73.9%	5651	6310	9553	6658	5255
3	T	2,008,050	306,556	15.3%	0	0	0	48,128	1
	R	496,184	330,078	66.5%	5321	7921	7767	9005	7113
4	T	1,326,216	734,294	55.4%	17,801	24,106	22,996	22,012	22,665
	R	772,552	465,740	60.3%	15,888	14,083	1	12,250	18,169
5	T	1,204,900	760,350	63.1%	28,945	13,913	16,688	12,844	16,613
	R	829,964	511,838	61.7%	8202	13,419	17,935	10,952	19,655
6	T	1,827,006	1,290,102	70.6%	28,549	47,391	36,286	41,191	39,938
	R	1,275,938	738,344	57.9%	8324	32,471	33,717	26,988	7109
7	T	976,420	330,748	33.9%	7517	7898	8576	13,420	8265
	R	1,408,788	557,530	39.6%	15,302	15,294	8648	27,698	10,507
8	T	2,983,738	1,071,686	35.9%	16,901	25,498	17,016	37,611	37,633
	R	1,669,418	1,111,106	66.6%	42,628	30,394	23,909	29,125	36,473
9	T	3,045,988	873,116	28.7%	18,814	31,957	20,327	20,666	19,411
	R	2,363,308	1,554,154	65.8%	49,812	37,580	43,342	32,717	42,284
10	T	2,940,066	837,538	28.5%	31,262	22,530	20,229	19,463	23,176
	R	529,382	308,928	58.4%	13,858	2	0	0	38,793
11	T	2,271,618	767,366	33.8%	18,698	25,124	19,429	16,500	26,844
	R	1,186,172	714,036	60.2%	29,603	28,473	19,391	9599	11,939
12	T	2,024,942	817,530	40.4%	10,914	36,474	8406	32,177	25,434
	R	1,016,076	347,702	34.2%	10,356	2518	9886	15,426	5253
13	T	2,486,864	925,342	37.2%	25,615	17,161	22,806	23,588	37,368
	R	2,363,774	1,373,208	58.1%	24,444	33,059	52,330	32,168	23,583
14	T	3,759,898	1,341,326	35.7%	40,059	0	40,757	39,834	34,610
	R	1,810,800	1,355,040	74.8%	46,339	13,972	45,339	44,861	27,602
15	T	1,317,164	412,320	31.3%	30,358	1	0	1	23,613
	R	968,980	325,430	33.6%	0	0	17,013	23,361	16,067
16	T	1,331,968	618,774	46.5%	29,254	22,446	32,310	0	6918
	R	630,316	120,392	19.1%	7314	9653	0	0	0
17	T	1,048,000	361,376	34.5%	7726	5205	4176	15,539	12,966
	R	670,036	282,286	42.1%	8027	10,852	16,204	13,602	0
18	T	1,532,694	750,230	48.9%	36,573	2	36,044	0	27,333
	R	1,987,742	1,121,518	56.4%	38,970	19,896	23,864	44,937	27,070
19	T	1,347,212	511,888	38.0%	15,749	9332	10,116	23,179	10,429
	R	967,458	173,226	17.9%	2566	8200	6438	3275	5148
20	T	955,798	359,270	37.6%	1	11,412	15,128	8055	15,936
	R	800,882	534,300	66.7%	0	15,343	0	23,908	32,984
21	T	1,480,150	408,196	27.6%	11,676	22,914	26,852	0	5194
	R	479,692	171,942	35.8%	9482	1580	1544	15,537	2159
22	T	1,878,320	853,640	45.4%	30,494	0	21,484	24,037	34,199
	R	1,461,076	289,924	19.8%	11,094	2	12,586	15,693	6607
23	T	1,299,418	439,690	33.8%	30,489	0	0	1	40,084
	R	459,734	130,094	28.3%	1352	2415	1584	1	13,040
24	T	1,734,882	415,668	24.0%	4491	9213	4739	46,431	3820
	R	784,992	291,082	37.1%	8732	15,432	7890	3910	4700
25	T	1,064,834	233,300	21.9%	5858	0	13,615	12,116	5768
	R	816,466	268,386	32.9%	7098	3232	9802	26,180	680
26	T	2,719,002	1,389,468	51.1%	34,784	52,475	25,991	21,910	31,423
	R	173,806	47,784	27.5%	1504	2302	1843	1902	1106
27	T	1,497,668	529,844	35.4%	42,978	1	0	0	27,274
	R	426,496	135,910	31.9%	7876	5620	4233	0	2993
28	T	1,410,508	447,986	31.8%	10,211	4865	31,045	1534	13,222
	R	570,904	127,652	22.4%	4729	1492	7213	5272	1786
29	T	1,978,482	884,750	44.7%	15,074	15,699	27,310	22,123	23,733
	R	413,578	163,606	39.6%	5288	5948	10,380	5455	2151
30	T	1,015,422	250,932	24.7%	3014	0	8113	21,758	2029
	R	406,356	126,946	31.2%	2871	6192	3261	3372	1554

Table S2-1. Sequencing statistics of the transmitter and recipient NGS data of all 30 pairs. The raw number of reads used for alignment and the number of reads in the final alignment are depicted in the second respectively third column. The fraction of aligned reads is shown in the fourth column. Finally, the average coverages for the five amplicons are shown in the last five columns.

S3 SeTesT: A statistical test for selection during transmission

We would like to detect changes in the composition of a virus population that occur during transmission from one host (the transmitter) to another (the recipient) and cannot be explained by random fluctuation alone. Such a situation may be indicative of selection acting on the virus population and may point to specific genotypes that are selected for or against during transmission. The difficulty in detecting selection during viral transmission lies in the large amount of random genetic drift that the transmitted virus population experiences initially, because typically only a very small number of virus particles are successfully transmitted. This transmission bottleneck creates strong random fluctuations that are difficult to distinguish from selective forces. It is critical to account for this effect in the testing procedure; otherwise the test would lead to hugely inflated false positive rates.

We model the transmission bottleneck explicitly as follows. We assume a very large (infinite) population size in the transmitter and a very small (finite) initial population size in the recipient. For transmission, we sample viruses from the transmitter population with probability proportional to their transmission fitness. Since the size of the recipient population is unknown, we account for this uncertainty by modelling the bottleneck size as an additional random variable.

The genomic composition of both the transmitter and recipient virus population have been assessed using next-generation sequencing (NGS). We regard these two sequencing read data sets as samples from the underlying genotype distributions of the respective virus populations. The test is based on comparing these distributions directly (rather than estimating model parameters). Significant differences between the two genotype distributions indicate deviation from neutrality during transmission, where the null distribution is designed in such a way that it accounts for variation due to the transmission bottleneck of unknown size.

In the following sections, we define the statistical test, called SeTesT (Selection Test in Transmission), formally and then assess its performance. We start by introducing a generative probabilistic model for the observed data, then we develop the test for a single locus of the viral genome and finally extend the framework to multi-locus genotypes. SeTesT is available at `https://github.com/cbg-ethz/SeTesT`.

S3.1 Probabilistic graphical model for read count data

Consider a single locus with a total number of K different observed genotypes. We first develop a probabilistic graphical model of the observed read count data and the underlying (unobserved) virus populations.

The virus population of the transmitter is assumed to be of infinite size. Hence it is described by a probability distribution over the K genotypes,

$$\boldsymbol{p} \in \Delta_{\mathbb{R}}^{K-1} := \left\{ (p_1, \ldots, p_K) \in \mathbb{R}_{>0}^K \;\middle|\; \sum_{k=1}^{K} p_k = 1 \right\} \tag{S3.1-I}$$

where p_k is the relative frequency of genotype k in the population. The virus population in the recipient is assumed to be of finite size $N > 0$ and defined as

$$\boldsymbol{Z} \in \Delta_{\mathbb{N}}^{K-1}(N) := \left\{ (Z_1, \ldots, Z_K) \in \mathbb{N}_{\geq 0}^K \;\middle|\; \sum_{k=1}^{K} Z_k = N \right\} \tag{S3.1-II}$$

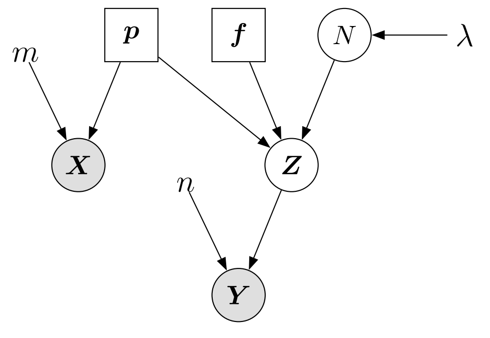

Figure S3.1-1. Probabilistic graphical model for observed genotype data. The transmitter read count data is the finite sample $\boldsymbol{X}$ with a total of m reads. The underlying virus population is $\boldsymbol{p}$. The finite virus population in the recipient is denoted by $\boldsymbol{Z}$ and an NGS sample $\boldsymbol{Y}$ with a total of n reads is drawn from it. The random variable N represents the unknown number of viruses transmitted and acts as a bottleneck parameter. If selective forces are absent, the fitness landscape $\boldsymbol{f}$ is flat and only stochastic forces shape the recipient virus population.

such that Z_k is the absolute frequency of genotype k in the population. The relative frequencies are denoted by $\boldsymbol{q} = \boldsymbol{Z}/N$. Both $\boldsymbol{p}$ and $\boldsymbol{Z}$ (and hence $\boldsymbol{q}$) are hidden random variables since we cannot observe the virus populations directly.

Since the total number of successfully transmitted viruses, N, is unknown, we model it as a random variable following a 0-truncated Poisson distribution with parameter λ,

$$N \sim \text{Pois}_{\setminus\{0\}}(\lambda) \tag{S3.1-III}$$

The rationale of this distribution lies in the fact that a very large population of viruses attempt to establish an infection, while only a very small mean number λ of viruses succeed. The distribution has positive support, because we condition on non-extinction, i.e., we know that an infection was established and that at least one virus had to be transmitted successfully.

We assume that the recipient virus population is the result of sampling virus particles with replacement from the transmitter population according to their transmission fitness. This amounts to a single-step Wright-Fisher process, a stochastic process with wide application in population genetics (Nowak, 2006), primarily to model the effects of genetic drift, i.e., random sampling in finite populations. Here, we employ it to model the effect of the transmission bottleneck.

We denote the fitness landscape by $\boldsymbol{f} \in \mathbb{R}^K_{>0}$. Since we model only relative fitness, we set $f_1 = 1$ without loss of generality. The probability of sampling genotype k is proportional to its relative frequency in the transmitter, p_k, and to its fitness, f_k. The transmission kernel $\boldsymbol{\psi}(\boldsymbol{p}, \boldsymbol{f})$ of the Wright-Fisher process is thus

$$\psi_k(\boldsymbol{p}, \boldsymbol{f}) = \frac{p_k f_k}{\sum_{l=1}^{K} p_l f_l}, \qquad k = 1, \ldots, K \tag{S3.1-IV}$$

and the successfully transmitted founder population $\boldsymbol{Z}$ is obtained as the multinomial sample

$$\boldsymbol{Z} \mid (\boldsymbol{p}, N, \boldsymbol{f}) \sim \text{Mult}(\boldsymbol{\psi}(\boldsymbol{p}, \boldsymbol{f}), N) \tag{S3.1-V}$$

Finally, we regard the observed NGS read count data as a finite sample of the corresponding virus population. For the transmitter, the m observed reads are modelled as

$$\boldsymbol{X} \mid (\boldsymbol{p}, m) \sim \text{Mult}(\boldsymbol{p}, m) \tag{S3.1-VI}$$

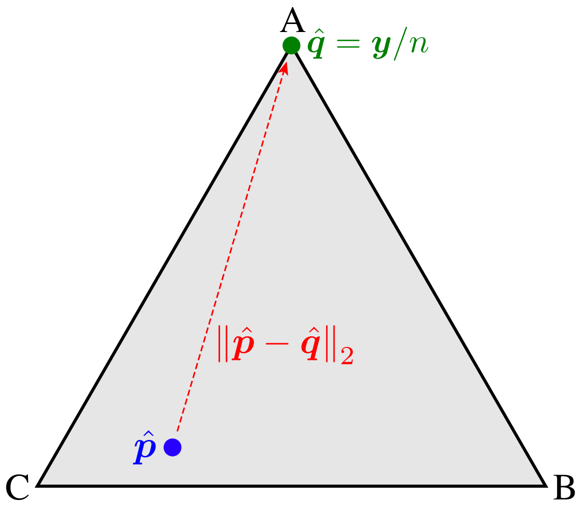

Figure S3.2-2. Construction of the test statistic. The simplex $\Delta^2_{\mathbb{R}}$ for the $K = 3$ genotypes A, B, and C is shown in light grey, with realisations of the transmitter virus population $\hat{\boldsymbol{p}}$ and the recipient virus population $\hat{\boldsymbol{q}}$. All three viral genotypes are present in the transmitter, but only genotype A in the recipient. This is due to fixation of A and extinction of B and C during transmission. The test statistic is the Euclidean distance between the two virus populations, with a realisation $t = \|\hat{\boldsymbol{p}} - \hat{\boldsymbol{q}}\|_2$ (dashed line). It captures the change the transmitted population has experienced. Once this change exceeds a critical threshold, the null hypothesis of neutral transmission is rejected, indicating that certain genotypes were selected for or against.

and similarly the n reads of the recipient as

$$\boldsymbol{Y} \mid (\boldsymbol{q}, n) \;\sim\; \text{Mult}\,(\boldsymbol{q},\, n) \tag{S3.1-VII}$$

The complete probabilistic graphical model for $(\boldsymbol{X}, \boldsymbol{Y})$ is defined by the parameters $\boldsymbol{p}$, $\boldsymbol{f}$ and λ in equations (S3.1-III)–(S3.1-VII) and summarised in Figure S3.1-1.

S3.2 Testing procedure

In the absence of selection, there are no fitness differences between the genotypes and hence they are sampled from the transmitter virus population with probability $\boldsymbol{p}$ as in a neutral Wright-Fisher process to found the recipient population. To detect deviation from neutrality, we define the test statistic

$$T := \|\boldsymbol{p} - \boldsymbol{q}\|_2 \tag{S3.2-VIII}$$

as the Euclidean distance between the virus populations in the transmitter and recipient. Thus, T measures the amount of genetic change experienced by the population moving through the transmission bottleneck (Figure S3.2-2). Large values of T indicate deviation from neutrality and that selective pressure has shaped the recipient virus population. In order to make such a call, we need to estimate T from the observed data and compare it to the distribution of T under the null hypothesis of neutrality.

Let $\boldsymbol{x}$ be an observation of $\boldsymbol{X}$, i.e., a collection of m genotypes (observed on NGS reads) sampled from the transmitter virus population, and similarly $\boldsymbol{y}$ an observation of $\boldsymbol{Y}$, i.e., a collection of n genotypes sampled from the recipient virus population. We estimate the relative genotype frequencies in the underlying populations as $\hat{\boldsymbol{p}} = \boldsymbol{x}/m$ and $\hat{\boldsymbol{q}} = \boldsymbol{y}/n$. The observed test statistic is then

$$t = \|\hat{\boldsymbol{p}} - \hat{\boldsymbol{q}}\|_2 \tag{S3.2-IX}$$

In order to assess the distribution of T under the null, we use the generative model from above (Figure S3.1-1) with a flat fitness landscape $\boldsymbol{f} = (1, \ldots, 1)$. We fix $\boldsymbol{p} \approx \hat{\boldsymbol{p}}$ throughout. This simplification ignores the uncertainty in the parameter estimate, but we show later that it is negligible for realistic values of λ and hence does not affect the type I error rate of the test. We draw samples of T as follows:

$$N \sim \text{Pois}_{\setminus\{0\}}(\lambda) \tag{S3.2-X}$$

$$\boldsymbol{Z} \sim \text{Mult}\,(\boldsymbol{p},\, N) \tag{S3.2-XI}$$

$$\boldsymbol{Y} \sim \text{Mult}\,(\boldsymbol{Z}/N,\, n) \tag{S3.2-XII}$$

$$T = \|\boldsymbol{p} - \boldsymbol{Y}/n\|_2 \tag{S3.2-XIII}$$

For a total of R Monte Carlo samples $T_1, \ldots, T_R$ obtained independently and identically in this manner, the p-value is approximated as

$$\frac{1}{R}\sum_{r=1}^{R} \mathbb{1}\left\{T_r \geq t\right\} \tag{S3.2-XIV}$$

which is the fraction of times the simulated value of T exceeds or is equal to the observed one.

S3.3 Estimating the mean number of founder viruses, λ

It remains to determine the parameter λ of the 0-truncated Poisson distribution (equations (S3.1-III) and (S3.2-X)). Instead of estimating λ from the data, we use prior information to set λ. Keele *et al.* (2008) analysed a cohort of 102 transmission pairs and showed that 78 HIV-1 infections by sexual transmission were most likely founded by exactly one virus. The remaining 24 infections were established by two or more viruses. Thus, we estimate the probability of a transmission being founded by exactly one virus to be $78/102$.

The 0-truncated Poisson distribution has the probability mass function

$$\text{Pois}_{\setminus\{0\}}(k;\lambda) = \frac{\text{Pois}(k;\lambda)}{1 - \text{Pois}(0;\lambda)}$$

and hence the probability of $k = 1$ virus being transmitted is $\lambda e^{-\lambda}/(1 - e^{-\lambda})$. We estimate λ by solving numerically the equation

$$\frac{78}{102} = \frac{\lambda e^{-\lambda}}{1 - e^{-\lambda}}$$

to find $\hat{\lambda} = 0.515$. We fix $\lambda = \hat{\lambda}$ throughout. The distribution $\text{Pois}_{\setminus\{0\}}(k;\hat{\lambda})$ has the expected value 1.78 meaning that we assume that the recipient virus population is founded on average by 1.78 virus particles.

S3.4 Multi-locus genotypes

The generative model and statistical test described above assume K different genotypes at a fixed genomic locus. We now focus on multi-locus genotypes. The number K of different genotypes will then generally be much higher. A significant practical complication arises due to divergent evolution occurring between the time of transmission and the time of sample collection. In multi-locus genotypes, the probability of a change occurring in this time period increases rapidly with the number of loci. The

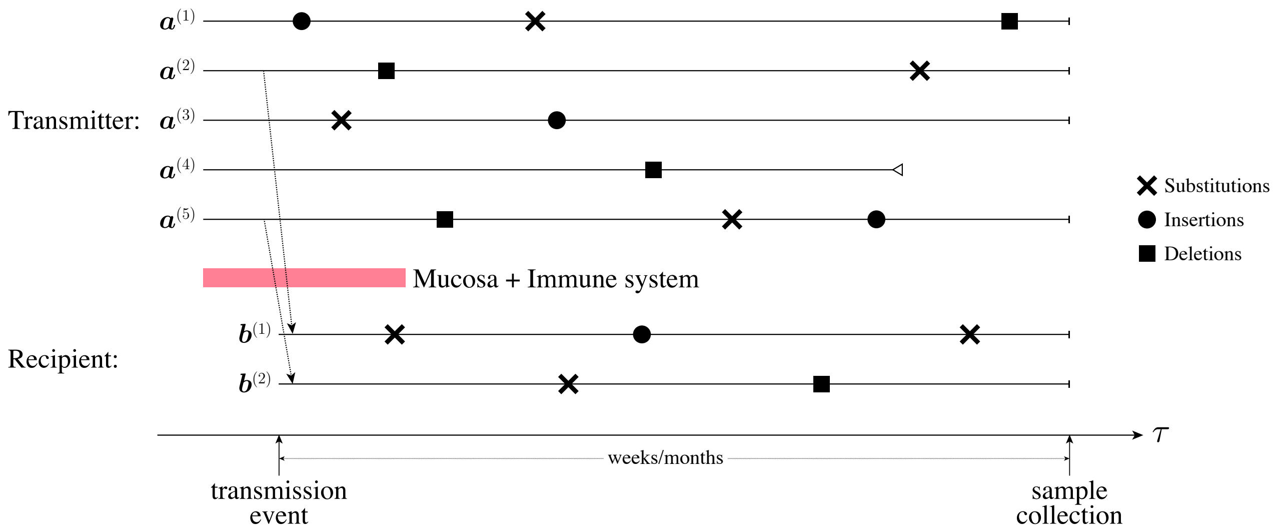

Figure S3.4-3. Illustration of divergence between transmitter and recipient. In this example, the transmitter harbours $K = 5$ genotypes. At some point, a transmission event occurs and the recipient receives virus of two different genotypes from the transmitter after having passed the recipient's mucosal membrane (pink) and evaded the immune system, denoted $\boldsymbol{b}^{(1)}$ and $\boldsymbol{b}^{(2)}$, which originated from $\boldsymbol{a}^{(2)}$ and $\boldsymbol{a}^{(5)}$, respectively. Meanwhile, weeks up to months can pass before such a transmission event is registered by the Swiss HIV Cohort Study and plasma samples can be collected. In this time, the intra-host HIV-1 populations in both transmitter and recipient diverge due to substitutions, insertions and deletions in their genomes, making a perfect match between the genotypes of transmitter and recipient unlikely. In addition, genotypes can go extinct due to intra-host evolution, such as is the case for genotype $\boldsymbol{a}^{(4)}$ in the transmitter, which goes extinct before sample collection.

observed transmitter and recipient multi-locus genotypes may therefore have very little exact overlap, and the test would not be applicable (Figure 4, S3.4-3).

In order to still be able to perform our test on such disjoint sets of sequences, we modify the test statistic T slightly and account for divergent evolution in the recipient. Our goal is to match recipient with transmitter multi-locus viral genotypes, such that their frequencies can again be compared using SeTesT. We consider a substitution model to measure divergence and take the uncertainty in the matching into account. In the following, we use a substitution model for amino acids, but similar models can be used for nucleotide substitutions in DNA sequences.

Let the genotypes $\boldsymbol{a}$, $\boldsymbol{b}$ be amino acid sequences of length L, where $\boldsymbol{a} = (a_1, \ldots, a_L)\,, a_i \in \mathcal{A}$ was observed in the transmitter and $\boldsymbol{b} = (b_1, \ldots, b_L)\,, b_i \in \mathcal{A}$ in the recipient, with $\mathcal{A}$ denoting the set of all 20 canonical amino acids. We assume the sequence sites to be independent and model the probability that the ancestor $\boldsymbol{a}$ has evolved into the descendent $\boldsymbol{b}$ in time τ as

$$P(\boldsymbol{b} \mid \boldsymbol{a}; \tau) = P(b_1, \ldots, b_L \mid a_1, \ldots, a_L; \tau) = \prod_{i=1}^{L} P(b_i \mid a_i; \tau) \qquad \text{(S3.4-XV)}$$

Employing Bayes' theorem we obtain for the probability of descendant $\boldsymbol{b}$ having as ancestor $\boldsymbol{a}$

$$P(\boldsymbol{a} \mid \boldsymbol{b}; \tau) = \frac{P(\boldsymbol{a}; \tau) \cdot P(\boldsymbol{b} \mid \boldsymbol{a}; \tau)}{P(\boldsymbol{b}; \tau)} = \prod_{i=1}^{L} \frac{\pi_{a_i} \cdot P(b_i \mid a_i; \tau)}{\pi_{b_i}} \qquad \text{(S3.4-XVI)}$$

where π and $P(b_i \mid a_i; \tau)$ will be defined below by a standard amino acid substitution model.

We estimate $\boldsymbol{q}$ now by considering for each recipient sequence all transmitter sequences as potential ancestors, weighted by the probability (S3.4-XVI). Let $\mathcal{X}$ and $\mathcal{Y}$ denote the set of sequences in the transmitter and recipient, respectively. The estimate of the recipient virus population is then

$$\hat{q}_{\boldsymbol{a}} = \frac{1}{n} \sum_{\boldsymbol{b} \in \mathcal{Y}} P\left(\boldsymbol{a} \mid \boldsymbol{b}; \tau\right) \, \boldsymbol{y}_{\boldsymbol{b}} \qquad \text{for all } \boldsymbol{a} \in \mathcal{X} \tag{S3.4-XVII}$$

It assigns the count mass $\boldsymbol{y}_{\boldsymbol{b}}$ of recipient genotype $\boldsymbol{b}$ to genotype $\boldsymbol{a}$ of the transmitter with probability $P\left(\boldsymbol{a} \mid \boldsymbol{b}; \tau\right)$, which we regard as the certainty in being able to match sequences. For multi-locus genotypes, we use the estimate (S3.4-XVII), rather than $\boldsymbol{y}/n$, in the test statistic (S3.2-IX).

We use the formulation by Nickle *et al.* (2007) as the basis for our substitution model. For the amino acid transition probabilities $\boldsymbol{P}(\tau) = [P(W \mid V;\, \tau)]_{V,W \in \mathcal{A}}$, we have

$$\frac{d}{d\tau} \boldsymbol{P}(\tau) = r \cdot \operatorname{diag}\left(\boldsymbol{\pi}\right) \boldsymbol{Q} \cdot \boldsymbol{P}(\tau) \tag{S3.4-XVIII}$$

where $\boldsymbol{Q}$ denotes the within-HIV-1 substitution matrix as estimated by Nickle *et al.* (2007). The vector $\boldsymbol{\pi} = \left(\pi_V\right)_{V \in \mathcal{A}}$ represents the equilibrium probability distributions for time $\tau \to \infty$, and r the average substitution rate per unit of time. The solution of this linear ordinary differential equation (ODE) is

$$\boldsymbol{P}(\tau) = \exp\left\{r \cdot \operatorname{diag}\left(\boldsymbol{\pi}\right) \boldsymbol{Q} \cdot \tau\right\} \tag{S3.4-XIX}$$

In practice, we employ an efficient numerical ODE solver as the spectral decomposition is usually not tractable. Furthermore, we constrain the parameters of the original matrix $\boldsymbol{Q}$ such that

$$\sum_{V \in \mathcal{A}} \pi_V \left[\operatorname{diag}(\boldsymbol{\pi})\boldsymbol{Q}\right]_{V,V} = -1$$

This translates into requiring the average substitution rate per unit of time to be 1.

As most recipients in our data are acute cases, they tend to harbour homogeneous viral populations. For this reason, we cannot, in general, estimate the average substitution rate r of (S3.4-XIX). Instead, we set $r = 4.6 \cdot 10^{-5}$ per day from Li *et al.* (2007) for amino acids.

It should be noted that substitution rates are only weakly related to mutation rates, for a number of reasons. The overall substitution rate is a product of many factors in the evolutionary process, such as fixation, synonymous and non-synonymous mutations, hitchhiking, recombination, etc. The mutation rate is a biochemical parameter characterising the viral reverse transcription process. Furthermore, both quantities have different units: substitution rate is a change per time interval, whereas mutation rate is a probability per replicated template base.

S3.5 Discussion of alternative statistical testing approaches

Given the nature of the observed data and the probabilistic model introduced above, at least two alternative strategies to test for departure from neutrality may be considered.

Tests for contingency tables. The observed genotype counts $(\boldsymbol{x},\, \boldsymbol{y})$ in the transmitter and recipient form a $2 \times K$ contingency table. As such, a significant difference between transmitter and recipient population could be detected by employing general tests for contingency tables, for example, Fisher's exact test or χ^2 tests. However, such an approach is insufficient: All general contingency table analysis ignores the extra variance introduced by the hidden random variables $\boldsymbol{Z}$ and N that account for the (strong) transmission bottleneck and would therefore massively inflate the false positive rate.

Likelihood ratio tests. In terms of our generative model (with $f_1 = 1$ fixed), the null hypothesis may be stated as $H_0 : \boldsymbol{f} = (1, \dots, 1)$, i.e., the fitness landscape is flat and during transmission the virus population is affected only by random sampling. The alternative hypothesis, namely that selection acts during transmission, is stated as $H_A : f_k \neq 1$ for at least one genotype $k \neq 1$. Testing this hypothesis directly requires tackling multiple challenges: First, there are no general solutions to statistical hypothesis testing in the presence of nuisance parameters. A popular method for testing in the presence of nuisance parameters is the likelihood ratio test. However, the asymptotic distribution of the likelihood ratio is well understood only if the maximum likelihood parameter estimates lie in the interior of the parameter space. In our situation, this will most often not be the case for the recipient virus population $\boldsymbol{q}$. Instead it will lie on a face of the probability simplex, because passing through the bottleneck $\boldsymbol{Z}$ will cause fixation and extinction of some of the genotypes.

S4 Validating the false positive rate

In order to judge whether a statistical test properly accounts for type I error rates, the distribution of p-values under H_0 is to be analyzed. A test yielding *valid* p-values is defined as $\mathbb{P}_\theta(p \leq \alpha) \leq \alpha$ for all possible values of α and $\theta \in \Theta$, where θ denotes some point of the parameter space Θ of the statistical model (Harrison, 2010). If strict inequality $\mathbb{P}_\theta(p \leq \alpha) < \alpha$ holds for some $\theta \in \Theta$, then the test is referred to as *conservative*, whereas if equality $\mathbb{P}_\theta(p \leq \alpha) = \alpha$ hold, the test is termed *exact*. On the other hand, if there exists some $\theta \in \Theta$ such that $\mathbb{P}_\theta(p \leq \alpha) > \alpha$ for some $\alpha \in [0, 1]$, then the test is referred to as *liberal* or *anti-conservative*. For statistical tests on discrete sample spaces, exact tests are usually impossible, due to the finite number of outcomes, which leads to discontinuities in the cumulative distribution function of the p-values. In such cases, it is recommended to err on the side of caution and prefer a more conservative test, as a conservative test fulfils our expectation of controlling the type I error rate set a priori by α (Rugg, 2007). In order to determine whether our developed statistical test yields valid p-values, we performed a number of simulations. Let K denote the number of genotypes, N_{sim} denote the number of simulations for a given set of parameter values $\boldsymbol{f} = (1, \ldots, 1)$, $\boldsymbol{p}$, m, n and λ. Unless otherwise stated, we have simulated each parameter set with $N_{\text{sim}} = 10\,000$ simulations.

S4.1 False positive rate for increasing λ

We first investigated the effect of the bottleneck parameter λ on the false positive rate. We drew the intra-host transmitter population $\boldsymbol{p}$ composed of $K = 2$ genotypes from a Dirichlet distribution with $\boldsymbol{\alpha} = (1, 1)^T$, which corresponds to a uniform distribution on $\Delta^1_{\mathbb{R}}$. It should be emphasised here that the correctness of the statistical test should be independent of the background distribution used to simulate intra-host viral populations. In our case, we opted for a Dirichlet distribution since it is the archetypal distribution on $\Delta^{K-1}_{\mathbb{R}}$ (Blei *et al.*, 2003). For each random $\boldsymbol{p}$, we simulate $\boldsymbol{X}$ and $\boldsymbol{Y}$ according to the graphical model in Figure S3.1-1 with $\lambda \in \{0.1, 0.5, 1, 10, 100, 10\,000\}$. We calculate the Monte Carlo based p-value using the equation (S3.2-XIV). Realisations of the simulations under H_0 are shown in Figure S4.1-1 for fixed n and m, and varying λ. For illustration purposes we have also analysed the resulting $\boldsymbol{X}$ and $\boldsymbol{Y}$ using Fisher's exact test, which is extremely anti-conservative in this context, as it does not account for the population bottleneck. For values of $\lambda \leq 100$, the distribution of approximate p-values is valid, that is, its ECDF lies on or below the identity function. For values of $\lambda \gg 100$ the distribution of approximate p-values starts to become anti-conservative, that is, $\mathbb{P}_\theta(p \leq \alpha) > \alpha$. This is due to the fact that for increasing λ, the bottleneck becomes weaker and at some point the uncertainty of the nuisance parameter $\boldsymbol{p}$ dominates the additional variance introduced by the Wright-Fisher bottleneck.

In order to gauge how strongly the conservativeness of the test influences the p-values, we analysed the tail of the p-value distribution more closely. By only looking at the empirical cumulative distribution function for a regime of significant $p < 0.05$ values, we could estimate by how much p-values are larger than necessary on average. With a linear fit we found that p-values are approximately inflated by a factor of 17.4 (Figure S4.1-2).

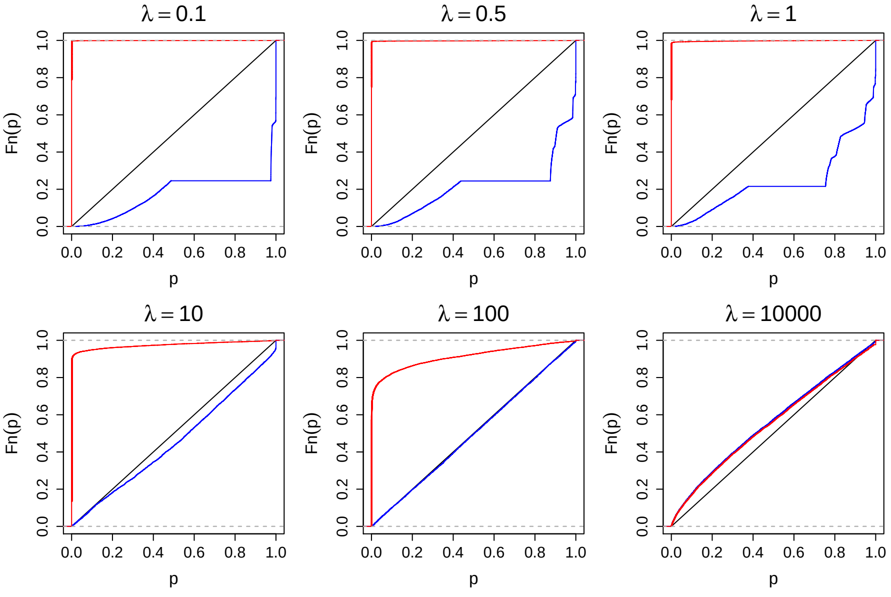

Figure S4.1-1. Empirical cumulative distribution function (ECDF) for null p-values for varying bottleneck sizes. We simulated from H_0 for various values of $\lambda \in \{0.1, \ldots, 10\,000\}$, where we fixed m and $n = 10\,000$. The x-axis of each plot denotes the value of the p-value, while the y-axis $\mathsf{Fn}(p)$ denotes the empirical value of the CDF for given p. The blue graph depicts the ECDF of p-values of our test. Below $\lambda = 100$ our test is always correct, whereas for extremely large values of λ the uncertainty in estimating $\boldsymbol{p}$ becomes the dominating factor and the test becomes anti-conservative. Notice how for realistic values of λ Fisher's exact test (red) tends to produce extremely small (incorrect) p-values.

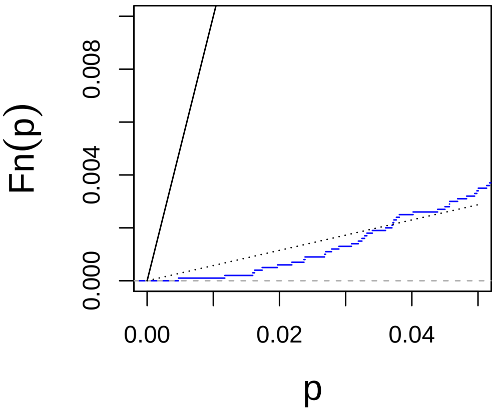

Figure S4.1-2. Estimating the conservativeness of the test for small p-values. The empirical cumulative distribution function (ECDF) for p-values smaller than 0.05 under H_0 for various values of $\lambda = 0.5$ is shown, where we fixed m and $n = 10\,000$. The x-axis of each plot denotes the value of the p-value, while the y-axis $\mathsf{Fn}(p)$ denotes the empirical value of the CDF for given p. The black solid graph depicts p-values under an exact test (i.e., the identity function $\mathsf{Fn}(p) = p$) and the blue graph depicts the ECDF of p-values of our test. The dotted line represents the best linear fit in the interval $p < 0.05$, with its slope estimated to be $1/17.4$.

S4.2 False positive rate for increasing transmitter coverage m

We performed further simulations, again with $K = 2$ and fixing $\lambda = 0.5$, the value of the bottleneck we expect in practice (subsection S3.3). We fixed n to $10\,000$ and varied m logarithmically from 10

to $10\,000$. The results of this simulation are depicted in Figure S4.2-3 and highlight how a coverage $m > 100$ does not affect the conservativeness of the test anymore.

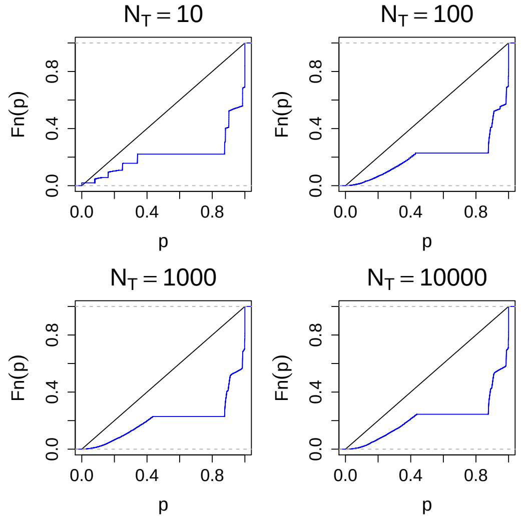

Figure S4.2-3. Empirical cumulative distribution function (ECDF) for null p-values for varying transmitter read coverages. We simulated from H_0 for various values of $m \in \{10, \ldots, 10\,000\}$, where we fixed $\lambda = 0.5, n = 10\,000$. The x-axis of each plot denotes the value of the p-value, while the y-axis $\mathsf{Fn}(p)$ denotes the empirical value of the CDF for given p. The blue graph depicts the ECDF of p-values of our test.

S4.3 False positive rate for increasing number of genotypes K

The final assessment of our test's false positive rate was conducted for increasing numbers of genotypes. We scaled K from 2 to 10, while fixing m and $n = 10\,000$ and $\lambda = 0.5$. With an increasing number of genotypes the test becomes less and less conservative. This is due to combinatorial number of different events that can occur, thereby decreasing the discreteness of the distribution of the test statistic.

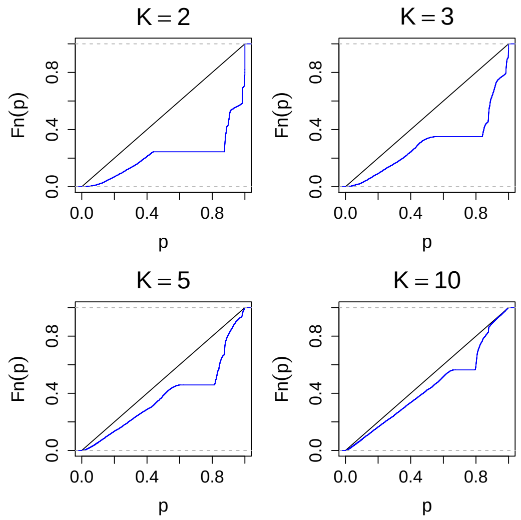

Figure S4.3-4. Empirical cumulative distribution function (ECDF) for null p-values for varying number of genotypes. We simulated from H_0 for various values of $K \in \{2, \ldots, 10\}$, where we fixed $\lambda = 0.5, m, n = 10\,000$. The x-axis of each plot denotes the value of the p-value, while the y-axis $\mathsf{Fn}(p)$ denotes the empirical value of the CDF for given p. The blue graph depicts the ECDF of p-values of our test.

S5 Sensitivity of detecting selection

Sensitivity, power or the true positive rate of a test is defined as $\mathbb{P}_\theta(p \leq \alpha \mid H_A)$, where H_A denotes the alternative hypothesis, i.e., selection acts on the population as the fitness landscape of the genotypes in the population is not constant. The sensitivity of the test will depend on a number of parameters: the significance level α, the selective coefficient s, the size of the bottleneck λ, the composition of the population $\boldsymbol{p}$ and the number of reads n and m. Given that α is fixed, we will not further investigate its effect on the sensitivity of the test. We analyse the sensitivity of our test when one genotype is being selected for and when one genotype is being selected against.

We simulated a population with selection acting at various strengths. To this end, we let the fitness f_1 of the first genotype vary on a logarithmic scale $f_1 \in [0.02, 50]$ and fix all other transmissibilities $f_j = 1$ for all $j \in \{2, \ldots, K\}$. We also vary the population composition, where we varied the frequency in the population of the first genotype p_1 in the interval $(0, 1)$ and set the value of all other genotypes' frequencies to $p_j = (1 - p_1) \,/\, (K - 1)$ for all $j \in \{2, \ldots, K\}$. We have fixed $m = 10\,000$ and $\lambda = 0.5$, in line with the average coverages we observed in the sequencing experiments and the bottleneck we estimated.

S5.1 Sensitivity for increasing recipient coverage n

We first assessed sensitivity of our test with increasing read coverage in the recipient. For this, we fixed $K = 2$ and the transmitter coverage $m = 10\,000$. We simulated data under different coverages for $n \in \{100, 1000, 10\,000\}$. The result of these simulations can be seen in Figure S5.1-1.

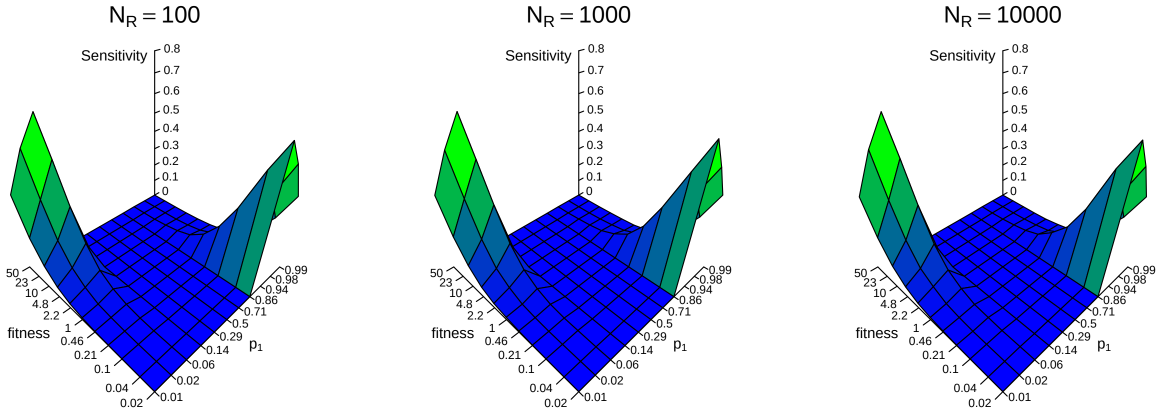

Figure S5.1-1. Sensitivity plots of the selection model for different read coverages in the recipient. The logit x-axis denotes the frequency of the first genotype p_1, the logarithmic y-axis denotes fitness f_1 of the first genotype and the z-axis depicts the sensitivity $\mathbb{P}(p \leq \alpha \mid p_1, f_1)$. At $f_1 = 1$ the null hypothesis H_0 holds and no selection acts. Notice the recurring phenomenon of the conservativeness of the test. In the interval $0.14 \leq p_1 \leq 0.86$, selection is not detectable at all, given that in this region, any fixation respectively extinction cannot generally yield p-values below $\alpha = 0.05$. All graphs are practically identical, i.e., sensitivity is not a function of n for the given range.

Sensitivity of our statistical test is not a function of the coverage in the recipient for $n \geq 100$. This is due to the population in the recipient generally being very homogeneous due to the strong bottleneck, with practically no variation or low frequency genotypes. HIV-1 in acute infections first undergoes clonal expansion and during this phase little diversity is present.

S5.2 Sensitivity for increasing number of genotypes K

We have simulated the model as in the previous section for, but this time increased the number of genotypes, starting at $K = 2$ going up to $K = 10$. We fixed $n, m = 10\,000$. While the picture is very similar, an increasing number of genotypes in the population to be tested does have a noticeable effect on our ability to detect selection (Figure S5.2-2). Detecting selection for a single genotype ($f_1 > 1$) is unchanged between $K = 2$ and $K = 10$. On the other hand, detecting a single genotype being selected against becomes easier with increasing number of genotypes. Notice how in the case of $K = 10$, we now have an increased chance of detecting selection in the parameter range $0.29 < p \leq 0.86$. This is explained by the fact, that in order to detect a single genotype being selected against, only one of the other $K - 1$ genotypes has to fixate, whereas to detect a single genotype being selected for, this, and only this genotype has to fixate. Due to the combinatorial advantage in the former, fixation of $K - 1$ other genotypes is probabilistically more likely and therefore easier to detect.

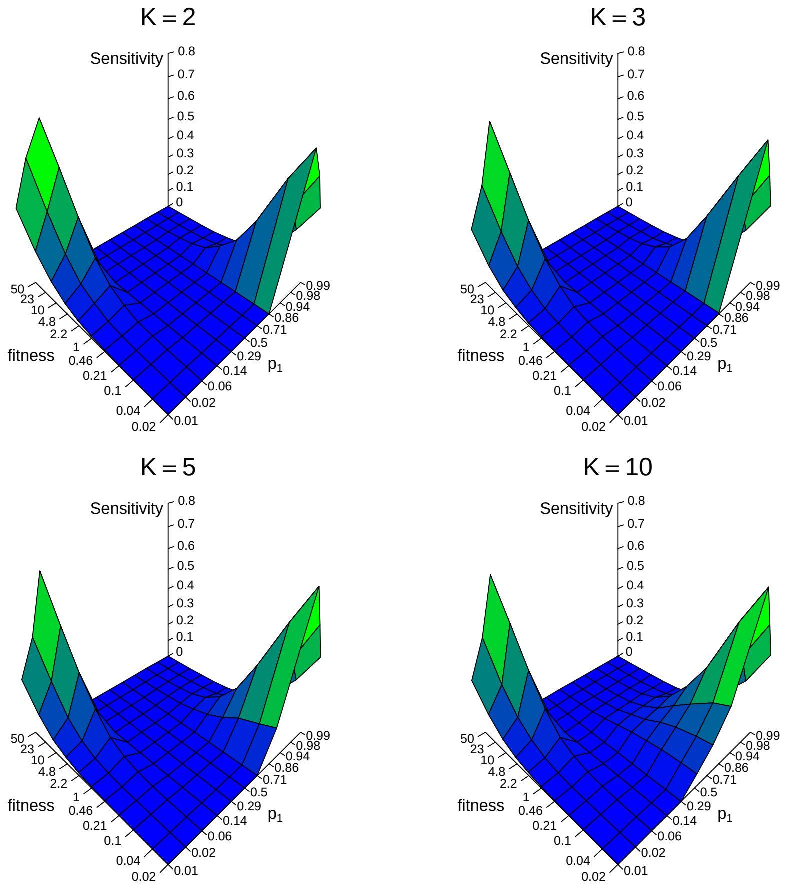

Figure S5.2-2. Sensitivity plots of the selection model for different K number of genotypes in the transmitter population. The logit x-axis denotes the frequency of the first genotype p_1, the logarithmic y-axis denotes fitness f_1 of the first genotype and the z-axis depicts the sensitivity $\mathbb{P}(p \leq \alpha \mid p_1, f_1)$. At $f_1 = 1$ the null hypothesis H_0 holds and no selection acts. In contrast to Figure S5.1-1, selection against a single genotype becomes detectable in an additional part of the parameter space.

S5.3 Increasing sensitivity by pooling patients

As the test has been shown to be generally conservative, with no statistical power in certain parameter ranges, we can combine independent p-values by using Fisher's method. Let N_{rep} denote the number of independent p-values, we then pool p-values by calculating $-2\sum_{i=1}^{N_{\text{rep}}}\ln(p_i)$ which is distributed according to a χ^2 distribution with $2 \cdot N_{\text{rep}}$ degrees of freedom. We simulated the sensitivity of our test when combining different number of p-values into one overall p-value. For this, we fixed $K = 2$, $n, m = 10\,000$ and varied the number of pooled p-values in the range $N_{\text{rep}} \in \{2, 3, 5, 10, 20, 30, 100, 1000, 10\,000\}$. We find that there is a stark gain in sensitivity in combining multiple patients (Figure S5.3-3), but only in certain parts of the parameter space. Even when combining the 30 p-values, we gain no power to detect selection for a single genotype when this genotype is abundant in the population. We consider transmission events as unusual when the majority genotype disappears, which will practically not occur when the genotype being selected for is already the majority genotype in the transmitter population.

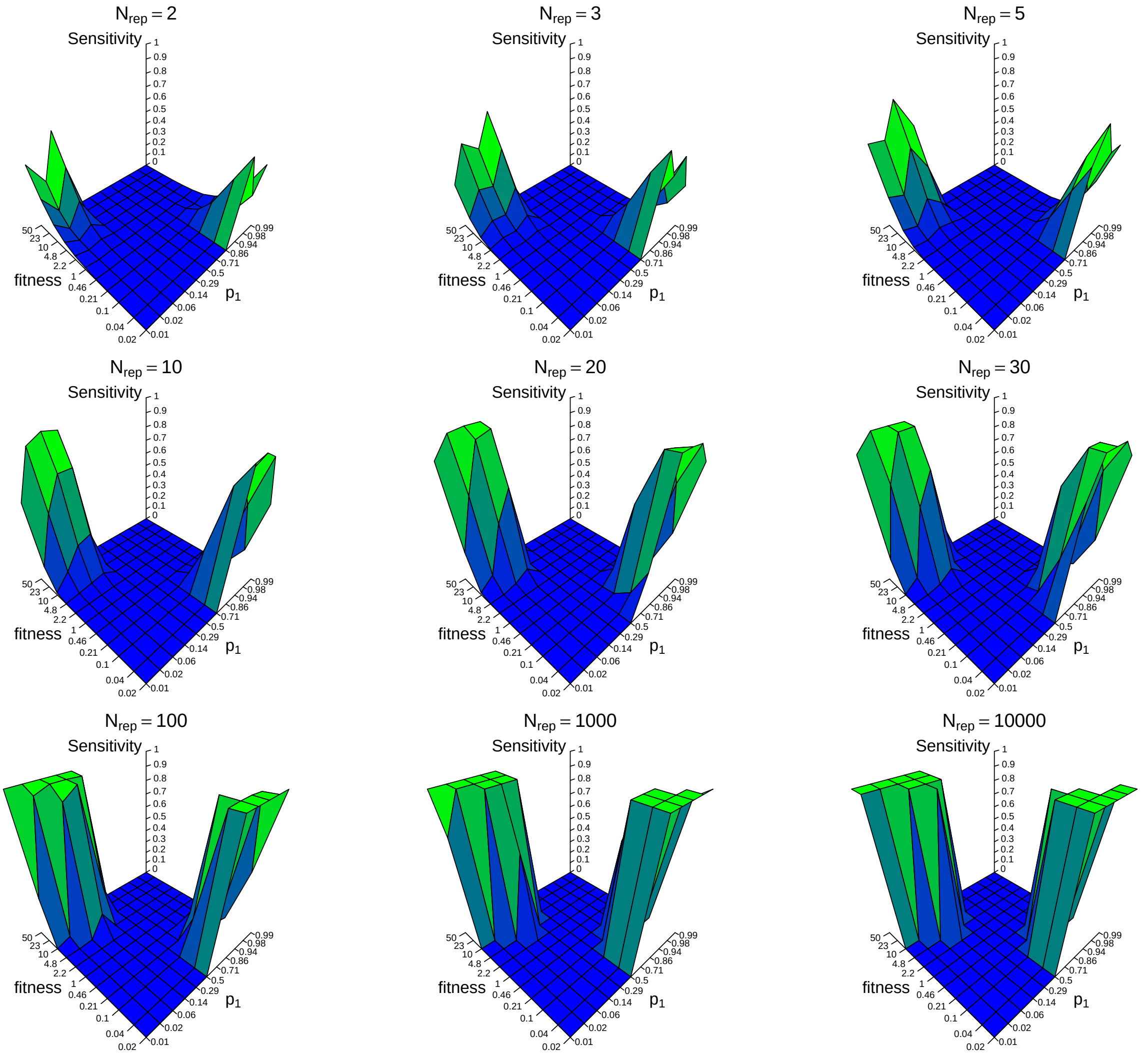

Figure S5.3-3. Sensitivity plots of the selection model for when combining the p-values of different N_{rep} number of pairs. The logit x-axis denotes the frequency of the first genotype p_1, the logarithmic y-axis denotes fitness f_1 of the first genotype and the z-axis depicts the sensitivity $\mathbb{P}(p \leq \alpha \mid p_1, f_1)$. At $f_1 = 1$ the null hypothesis H_0 holds and no selection acts. Statistical sensitivity approaches 1.0 close to the boundaries of the parameter space, yet no gains in power are apparent in other regions.

S6 Heatmaps and number of genotypes across the HIV-1 genome

We tested 2773 amino acid loci of all complete reading frames of HIV-1, where at least six or more tests could be performed across all 30 patients. We removed some regions of *gp120*, due in part to hypervariable regions not aligning well that could lead to spurious calls. We omitted analysing *rev* and exon 2 of *tat* as they are wholly contained within *gp120*.

Reading Frame	Number of analysed (total) amino acids	Genomic offsets
p17	132	790 – 1186
p24	231	1186 – 1879
p2-p7-p1-p6	137	1879 – 2290
Protease	99	2253 – 2550
RT	440	2550 – 3870
RNase	120	3870 – 4230
Integrase	288	4230 – 5094
vif	192	5041 – 5617
vpr	96	5559 – 5771 (frameshift insertion) 5772 – 5848
tat (exon 1)	71	5831 – 6044
vpu	82	6062 – 6308
gp120	334 (458)	6315 – 6615 (V1/V2 loop) 6813 – 7110 (V3 loop) 7218 – 7377 (V4 loop) 7479 – 7602 (V5 loop) 7635 – 7758
gp41	345	7758 – 8793
nef	206	8797 – 9415

Table S6-1. Reading frames with offsets with respect to HXB2. Note that some parts of reading frames have been omitted, for various practical reasons. All genomic indices are half-open intervals, meaning the first position is included, but not the last position.

A number of patients had failed amplicons during sample preparation, which manifest as large vertical bars in the paired transmitter and recipient amino acids plots (Figure S6-2 – S6-28).

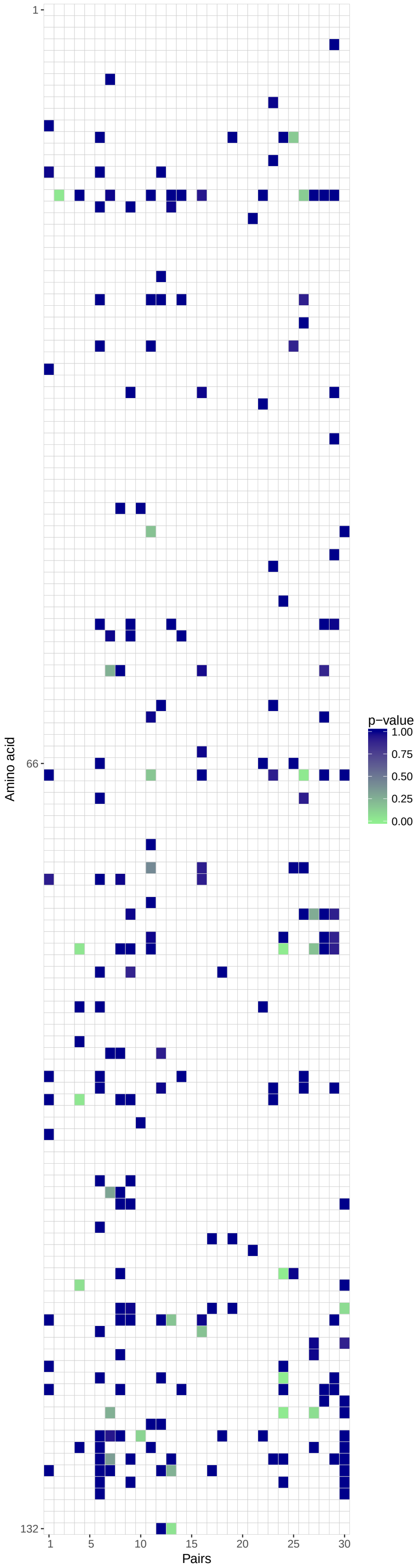

Figure S6-1. Heatmaps of p-values for the *p17* reading frame. Each column represents the test outcomes for a transmitter-recipient pair and every row represents one amino acid locus out of 132 across all 30 recipient and transmitters.

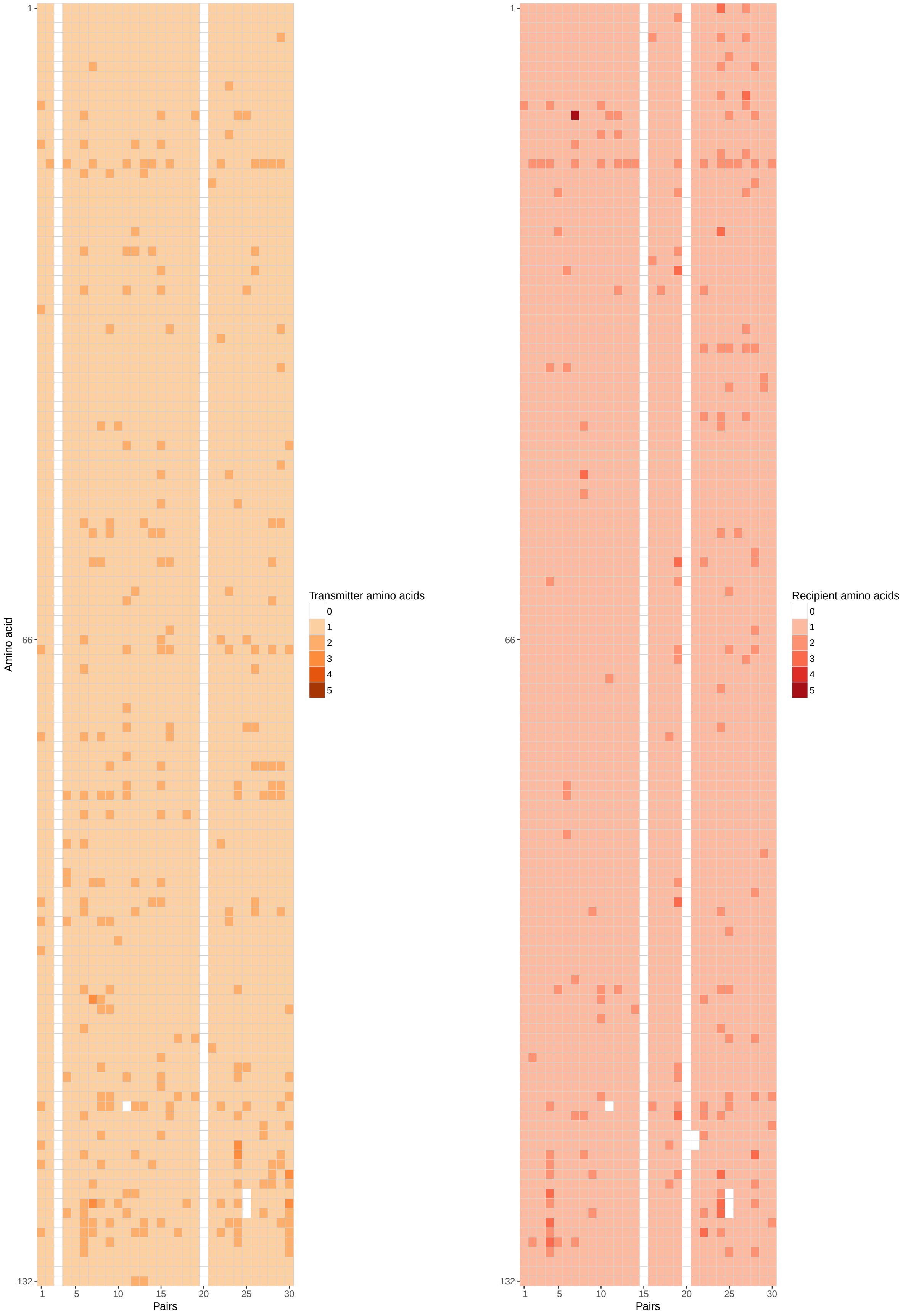

Figure S6-2. Heatmaps of individual amino acid counts for the *p17* reading frame. The left respectively right heatmap shows the number of amino acids in the transmitter, respectively in the recipient at the analysed 132 positions across all 30 recipient and transmitters.

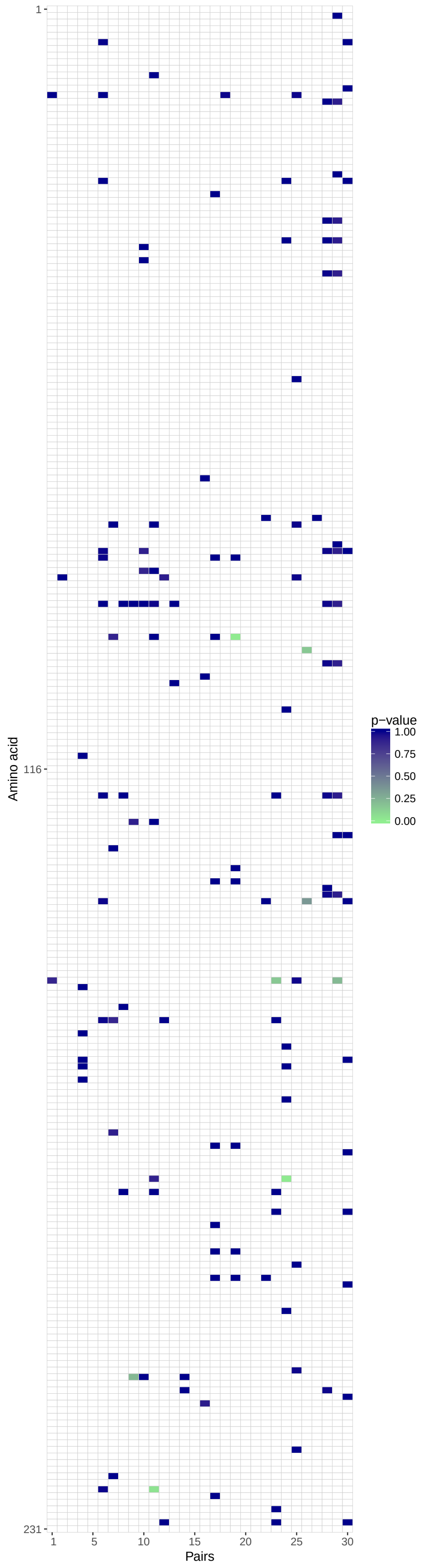

Figure S6-3. Heatmaps of p-values for the *p24* reading frame. Each column represents the test outcomes for a transmitter-recipient pair and every row represents one amino acid locus out of 231 across all 30 recipient and transmitters.

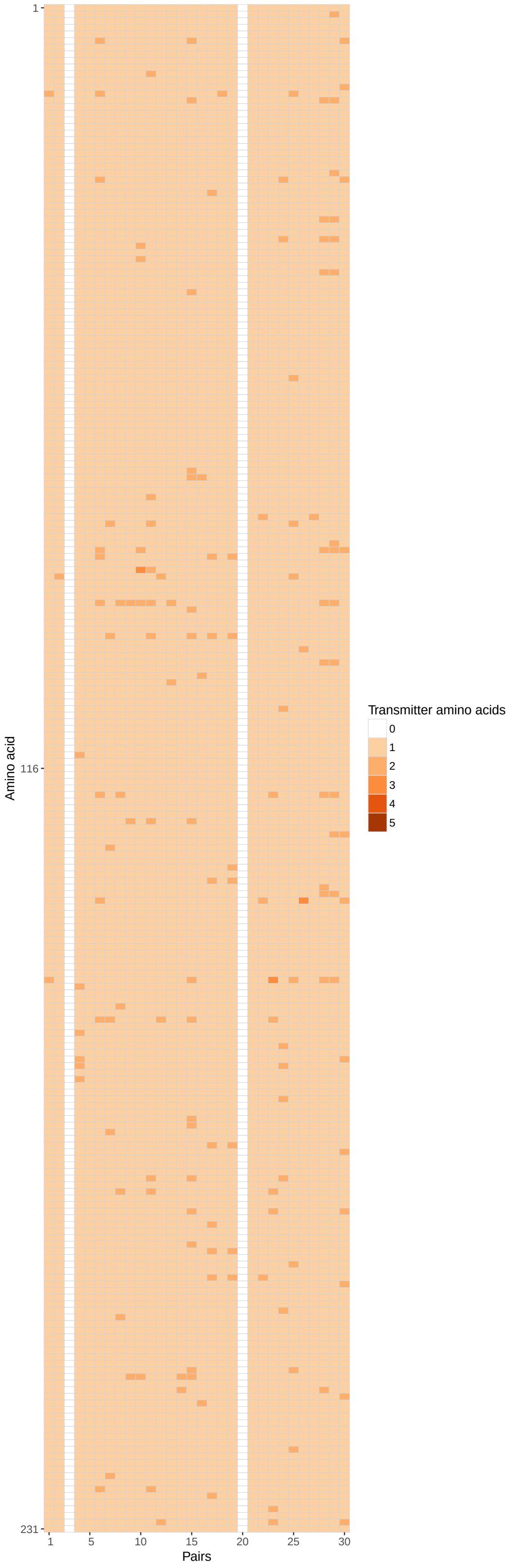

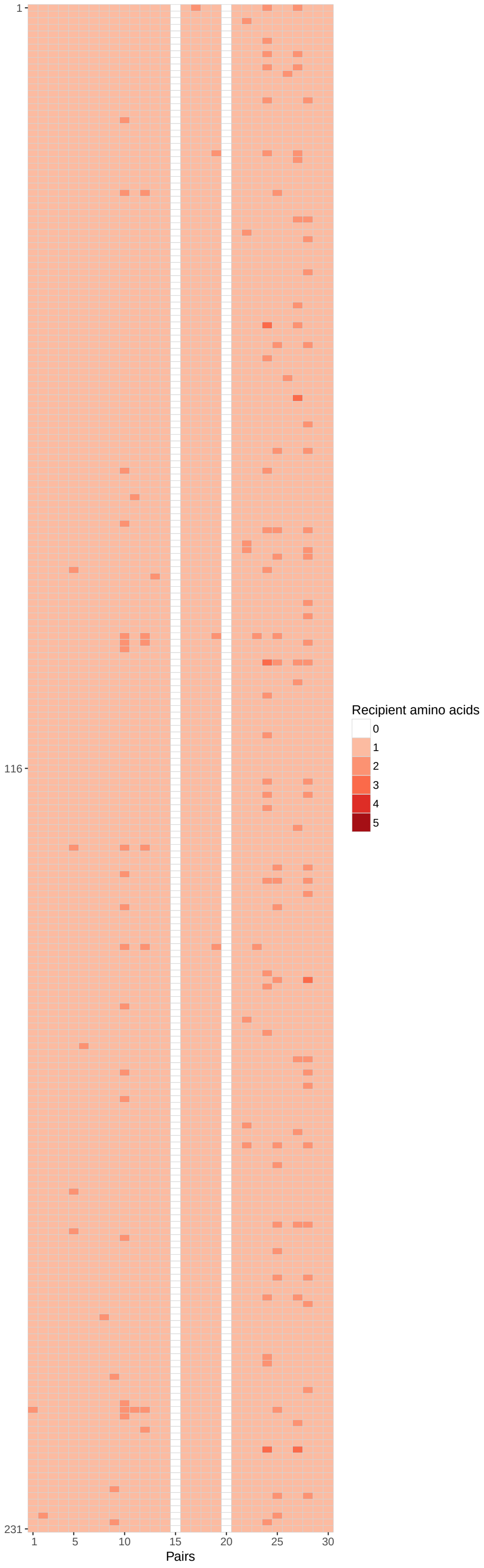

Figure S6-4. Heatmaps of individual amino acid counts for the *p24* reading frame. The left respectively right heatmap shows the number of amino acids in the transmitter, respectively in the recipient at the analysed 231 positions across all 30 recipient and transmitters.

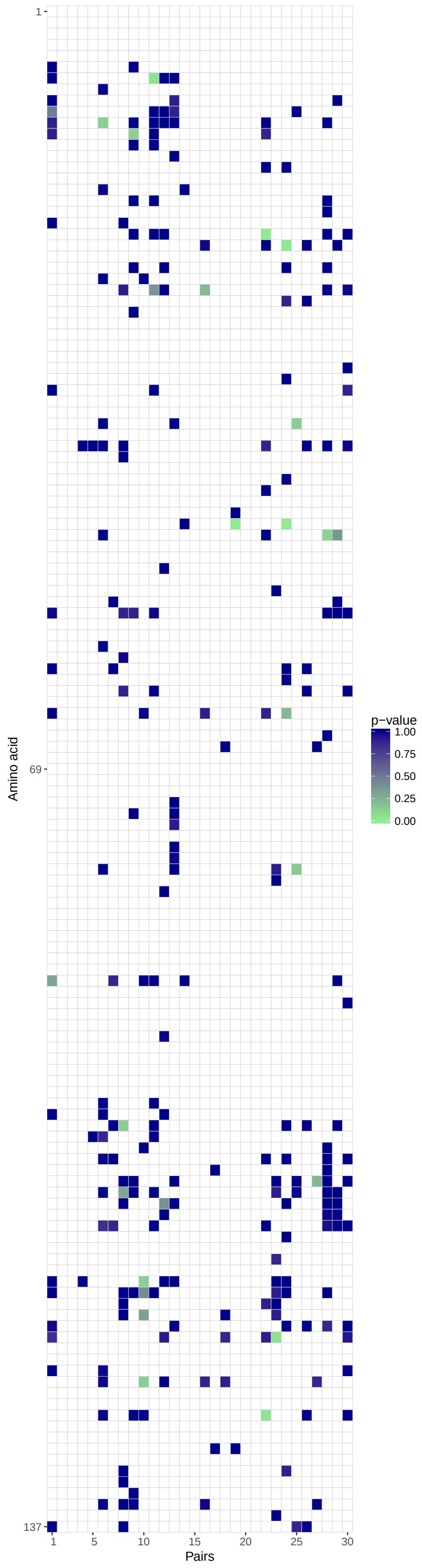

Figure S6-5. Heatmaps of p-values for the *p2-p7-p1-p6* reading frame. Each column represents the test outcomes for a transmitter-recipient pair and every row represents one amino acid locus out of 137 across all 30 recipient and transmitters.

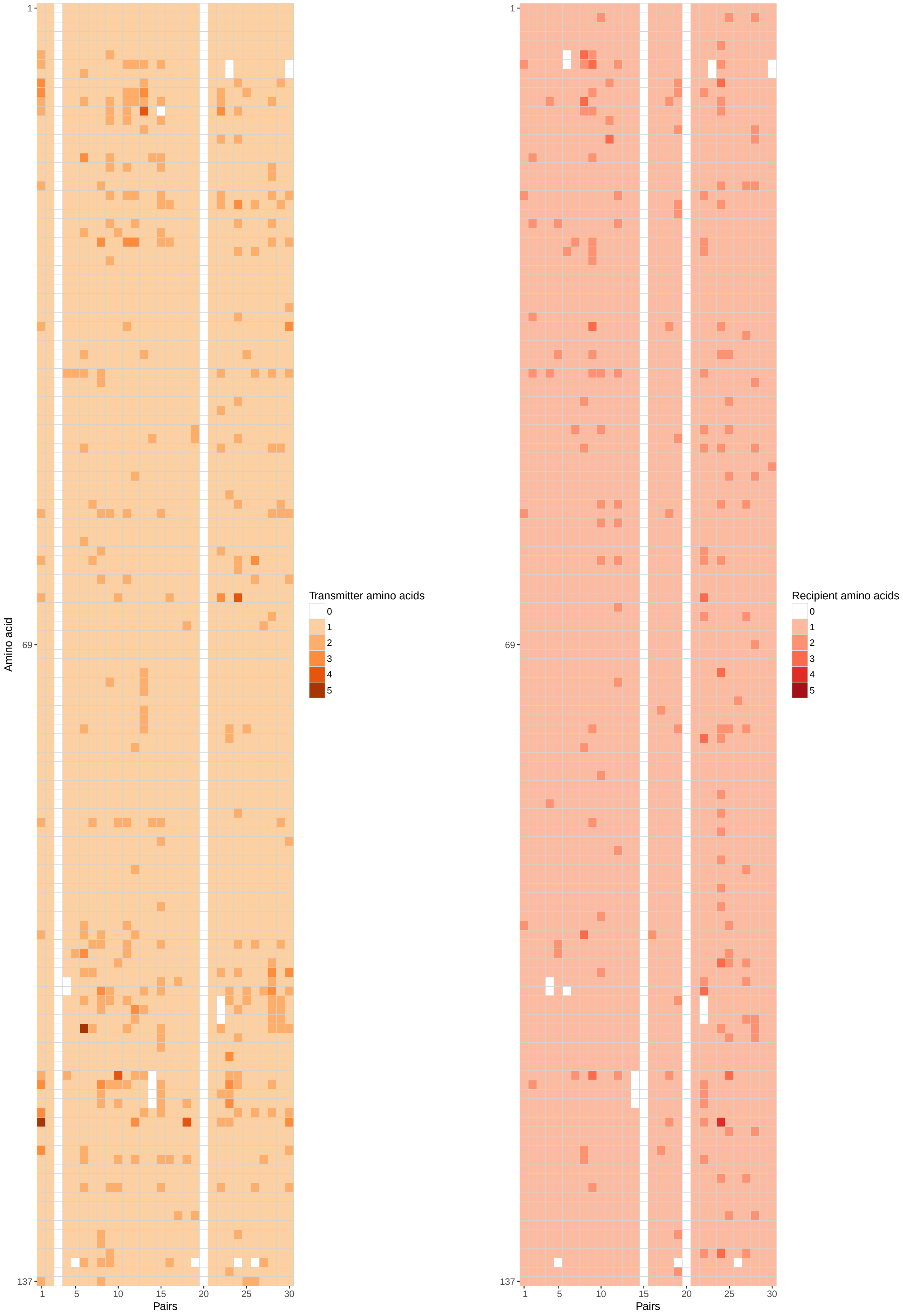

Figure S6-6. Heatmaps of individual amino acid counts for the *p2-p7-p1-p6* reading frame. The left respectively right heatmap shows the number of amino acids in the transmitter, respectively in the recipient at the analysed 137 positions across all 30 recipient and transmitters.

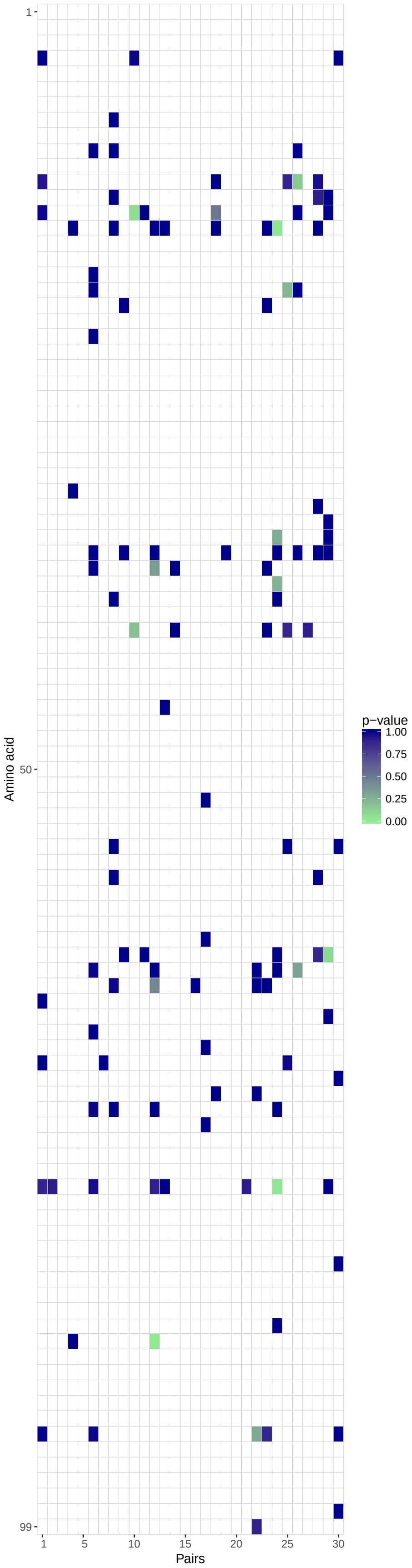

Figure S6-7. Heatmaps of p-values for the *Prot* reading frame. Each column represents the test outcomes for a transmitter-recipient pair and every row represents one amino acid locus out of 99 across all 30 recipient and transmitters.

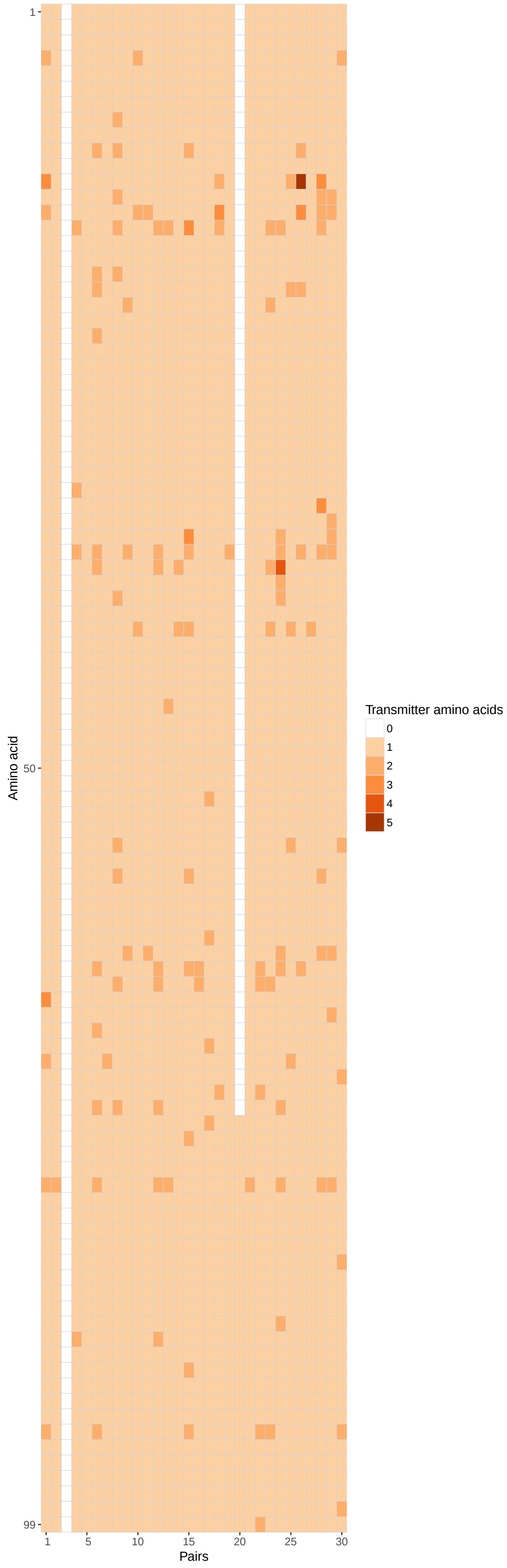

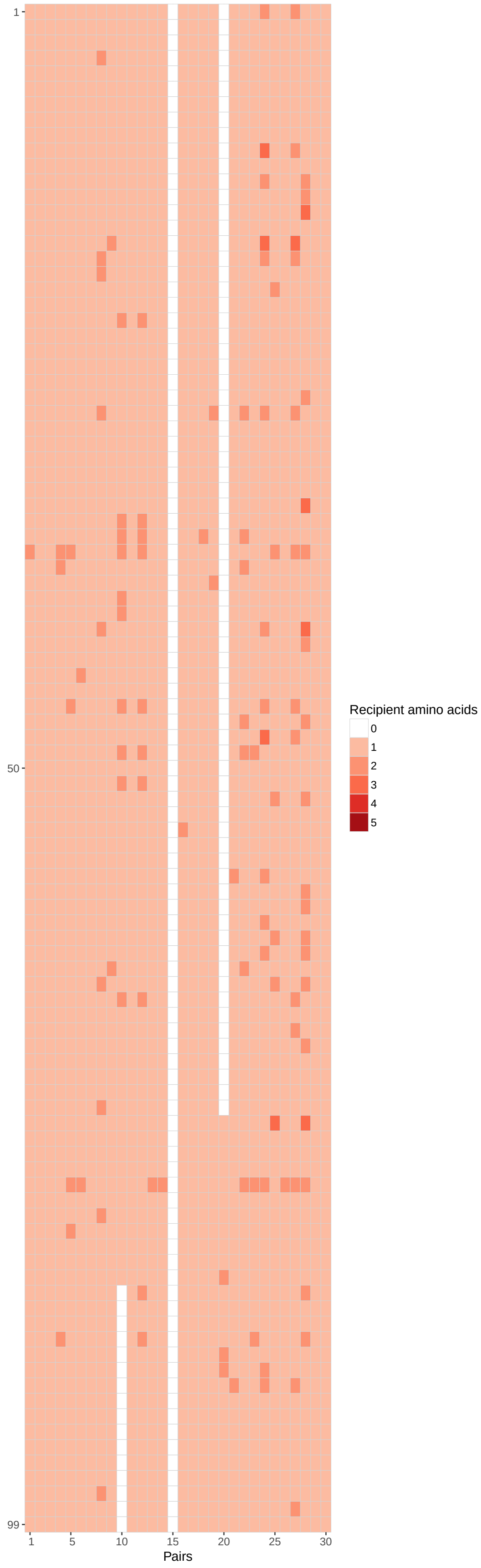

Figure S6-8. Heatmaps of individual amino acid counts for the *Prot* reading frame. The left respectively right heatmap shows the number of amino acids in the transmitter, respectively in the recipient at the analysed 99 positions across all 30 recipient and transmitters.

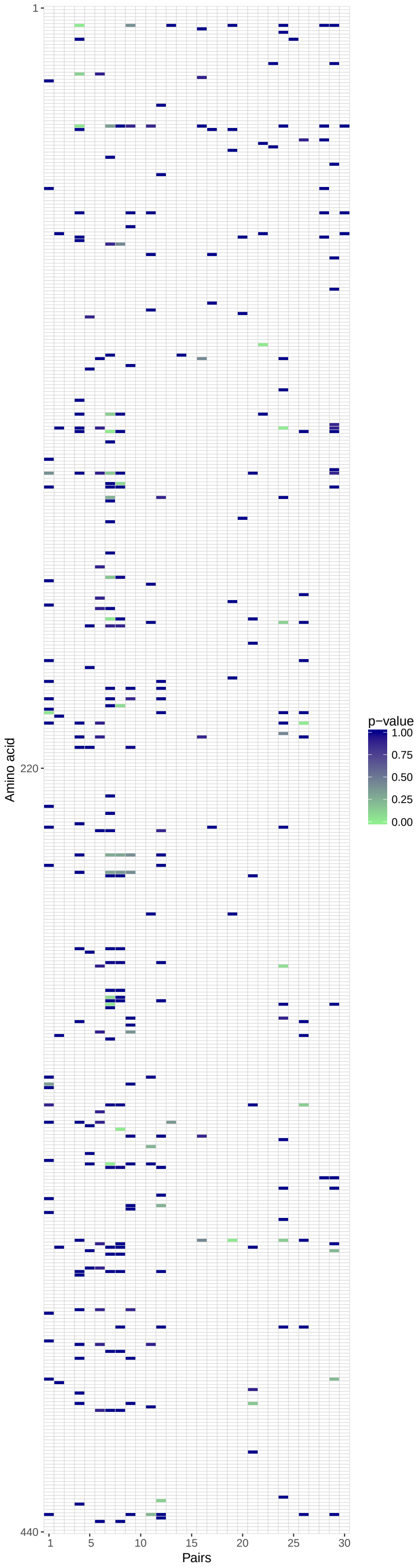

Figure S6-9. Heatmaps of p-values for the *RT* reading frame. Each column represents the test outcomes for a transmitter-recipient pair and every row represents one amino acid locus out of 440 across all 30 recipient and transmitters.

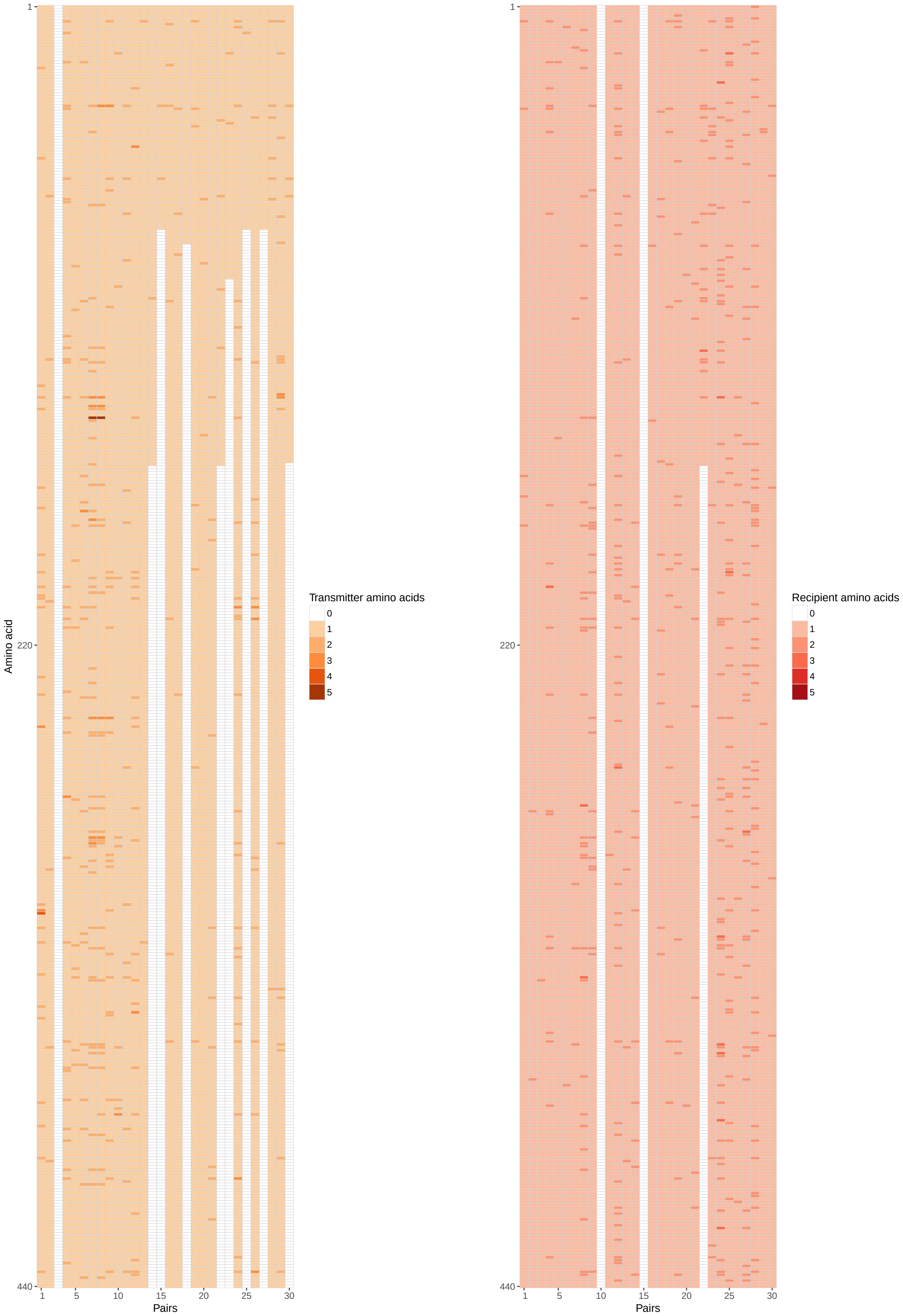

Figure S6-10. Heatmaps of individual amino acid counts for the *RT* reading frame. The left respectively right heatmap shows the number of amino acids in the transmitter, respectively in the recipient at the analysed 440 positions across all 30 recipient and transmitters.

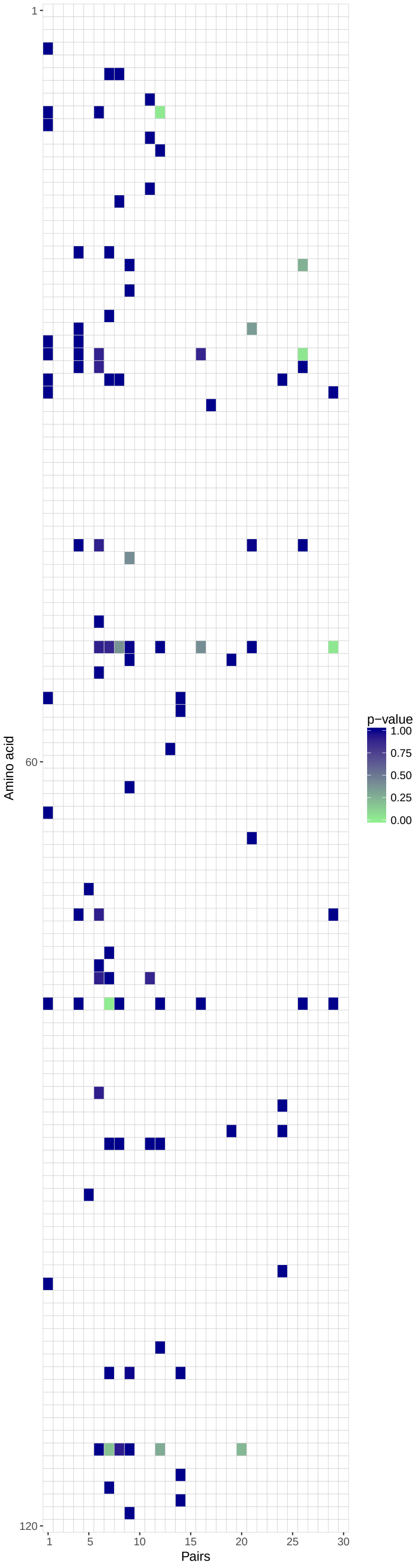

Figure S6-11. Heatmaps of p-values for the *RNase* reading frame. Each column represents the test outcomes for a transmitter-recipient pair and every row represents one amino acid locus out of 120 across all 30 recipient and transmitters.

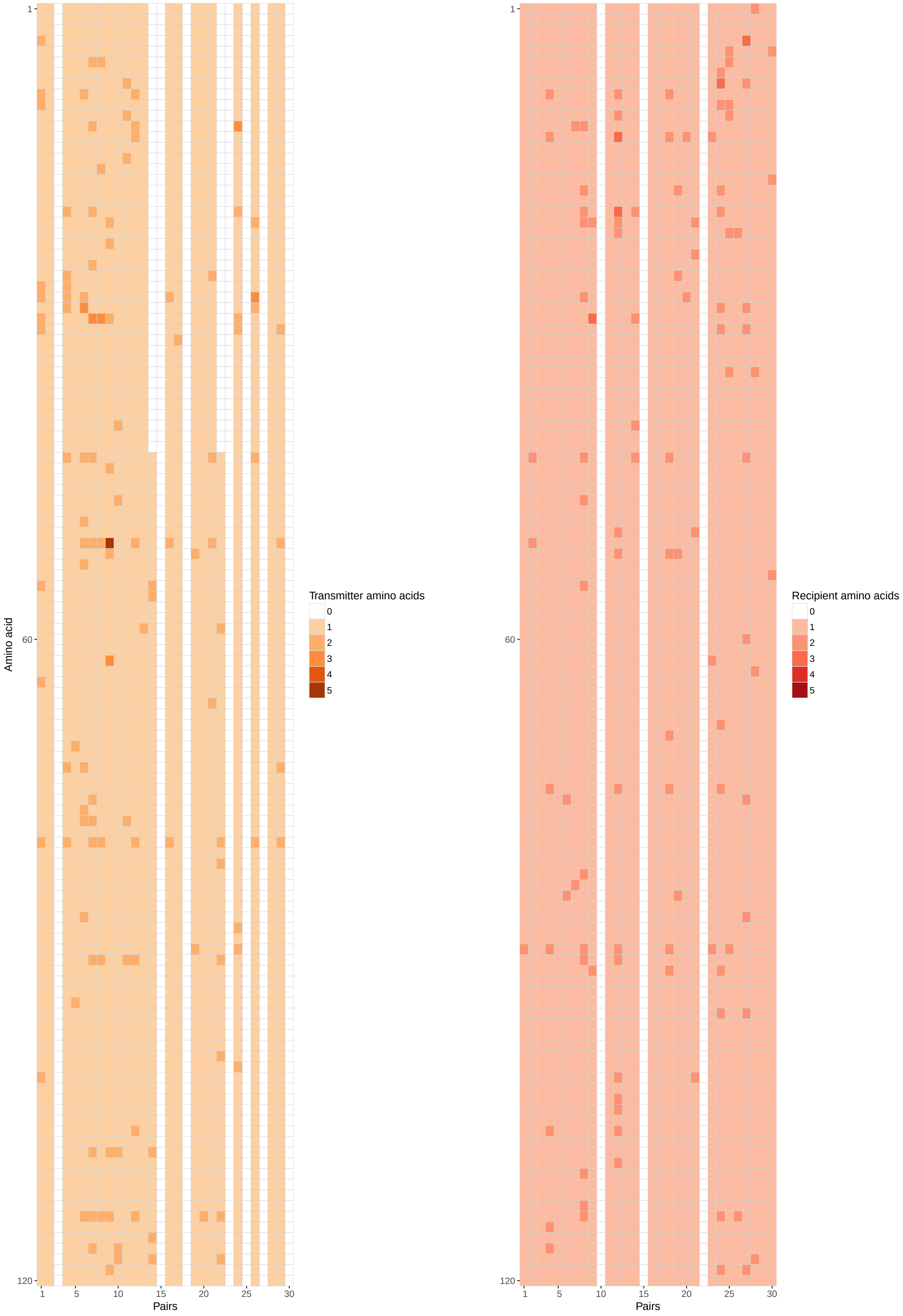

Figure S6-12. Heatmaps of individual amino acid counts for the *RNase* reading frame. The left respectively right heatmap shows the number of amino acids in the transmitter, respectively in the recipient at the analysed 120 positions across all 30 recipient and transmitters.

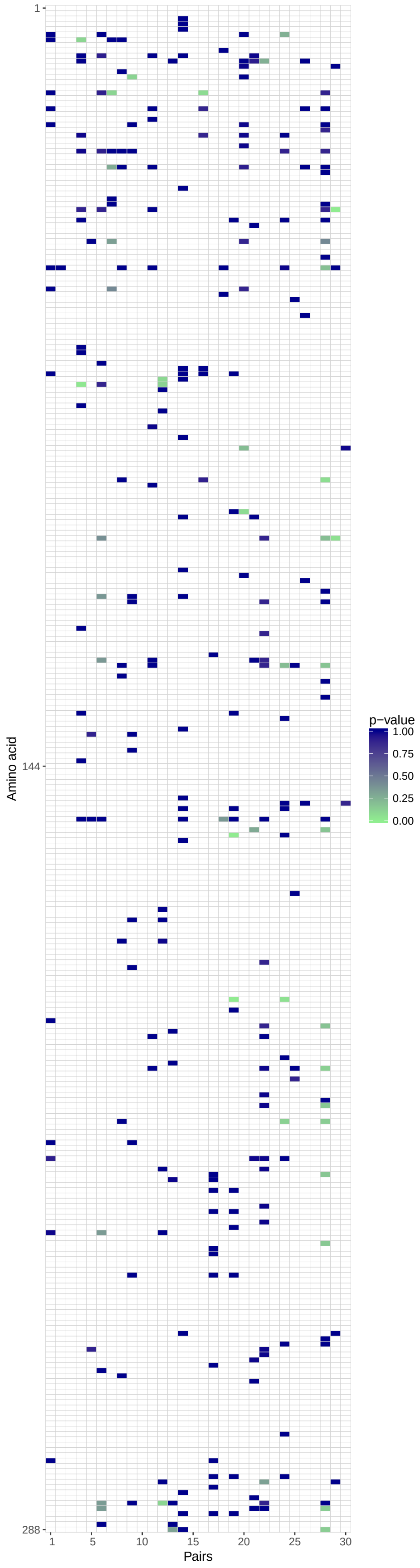

Figure S6-13. Heatmaps of p-values for the *Int* reading frame. Each column represents the test outcomes for a transmitter-recipient pair and every row represents one amino acid locus out of 288 across all 30 recipient and transmitters.

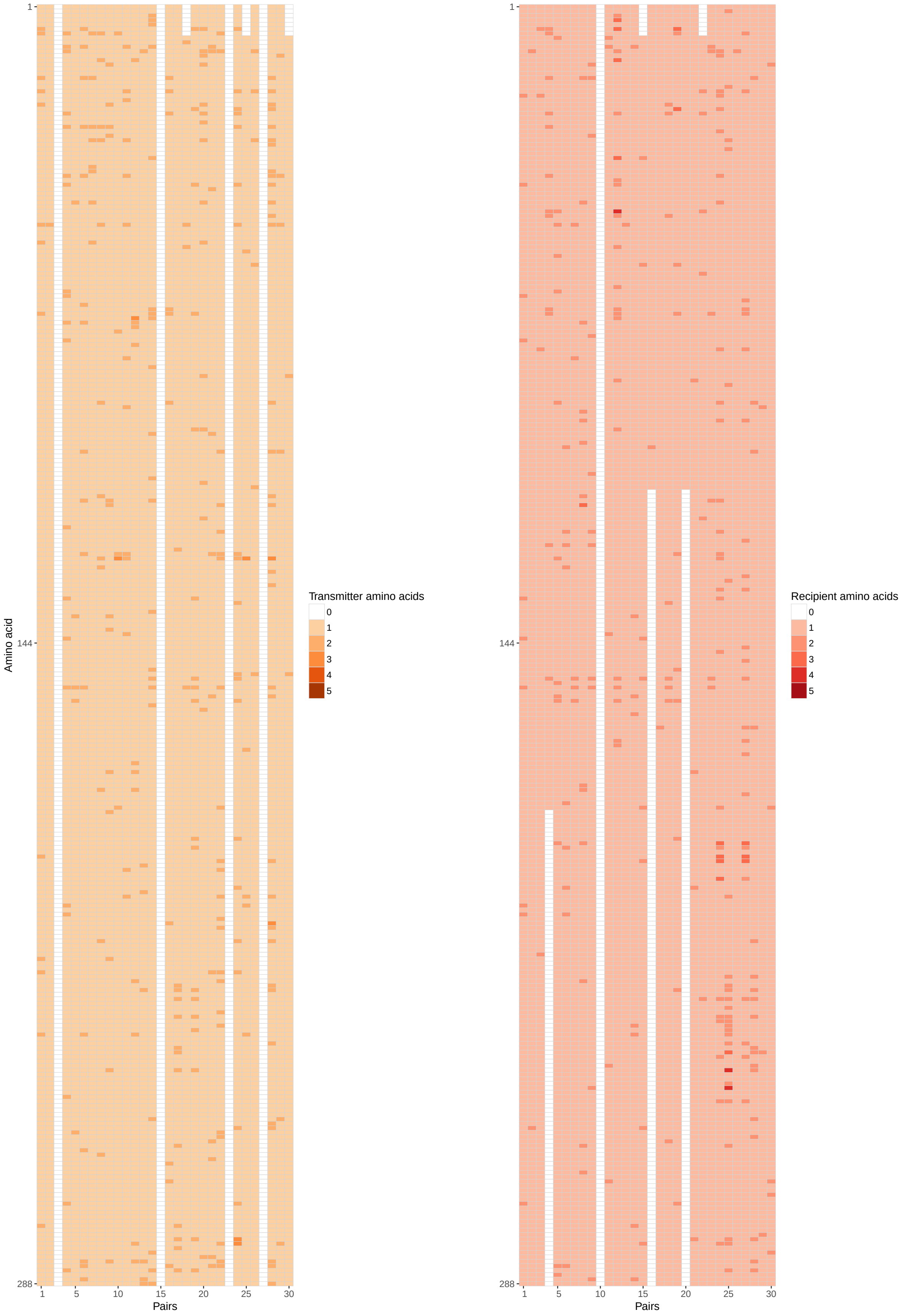

Figure S6-14. Heatmaps of individual amino acid counts for the *Int* reading frame. The left respectively right heatmap shows the number of amino acids in the transmitter, respectively in the recipient at the analysed 288 positions across all 30 recipient and transmitters.

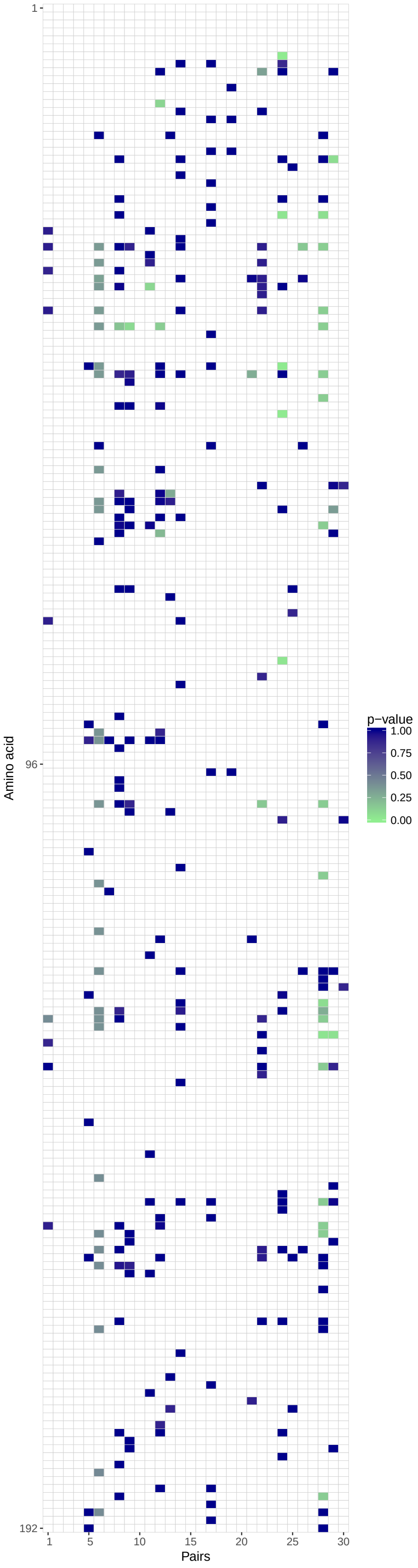

Figure S6-15. Heatmaps of p-values for the *vif* reading frame. Each column represents the test outcomes for a transmitter-recipient pair and every row represents one amino acid locus out of 192 across all 30 recipient and transmitters.

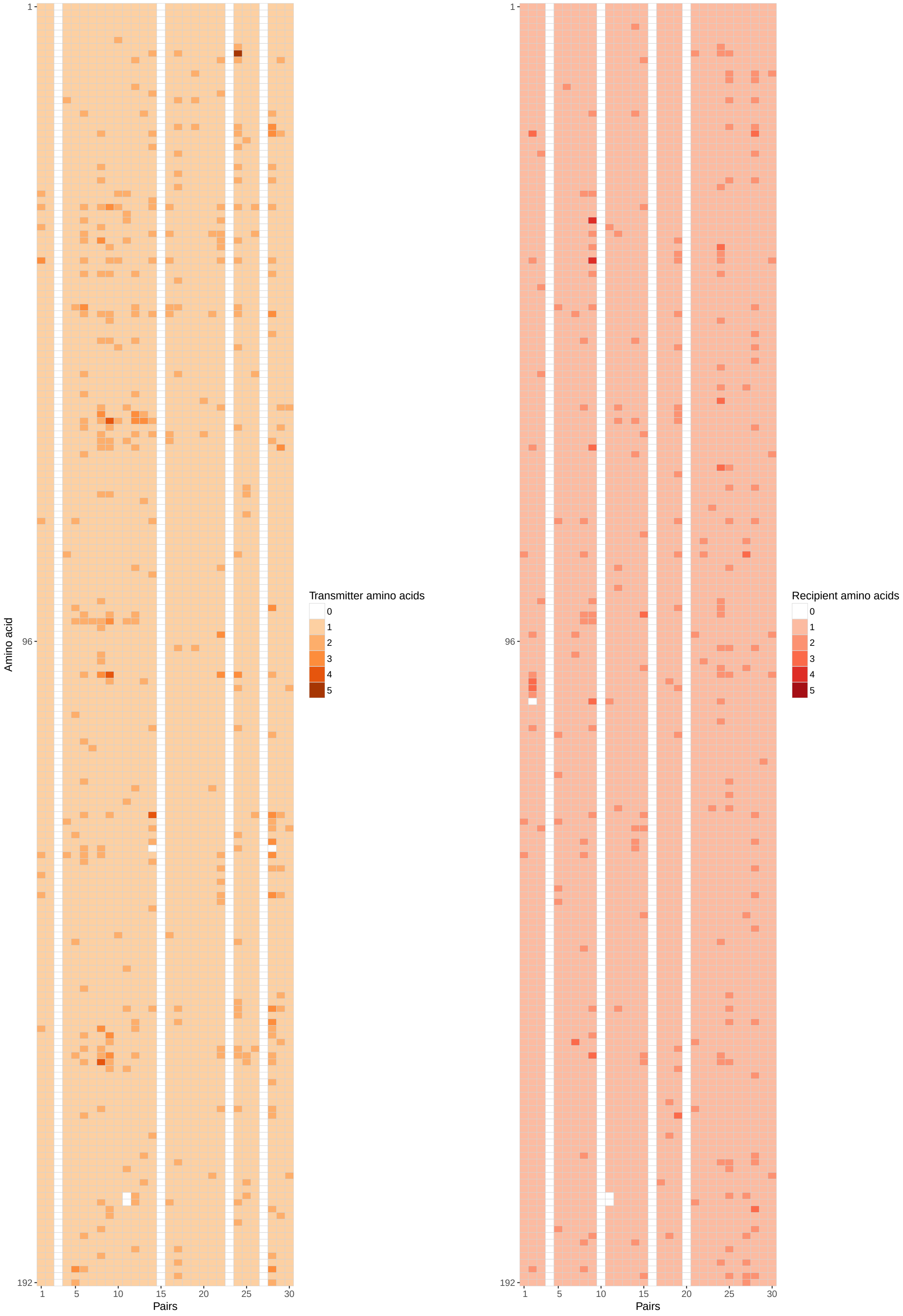

Figure S6-16. Heatmaps of individual amino acid counts for the *vif* reading frame. The left respectively right heatmap shows the number of amino acids in the transmitter, respectively in the recipient at the analysed 192 positions across all 30 recipient and transmitters.

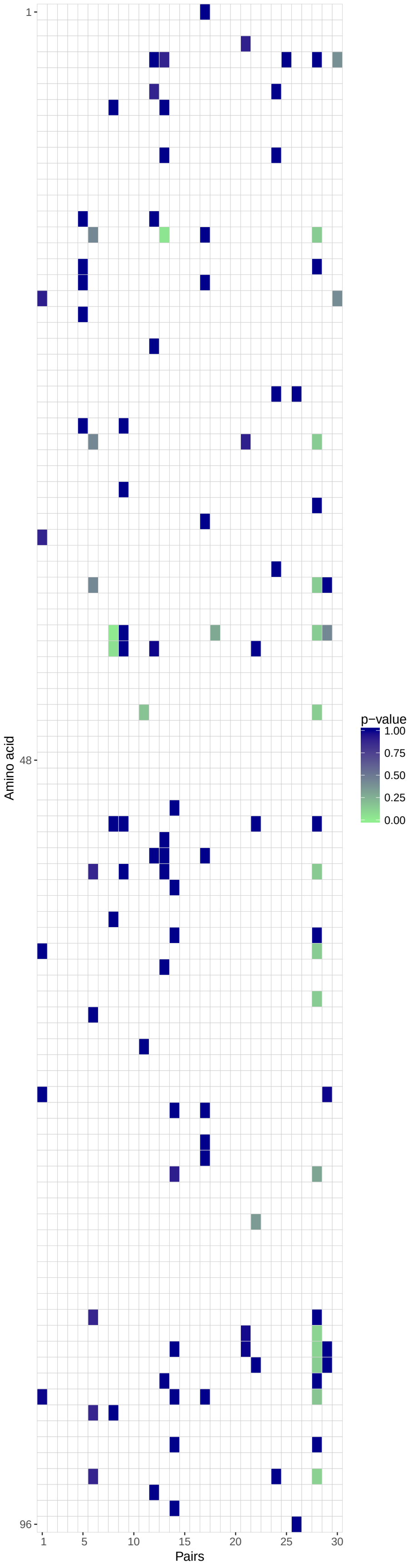

Figure S6-17. Heatmaps of p-values for the *vpr* reading frame. Each column represents the test outcomes for a transmitter-recipient pair and every row represents one amino acid locus out of 96 across all 30 recipient and transmitters.

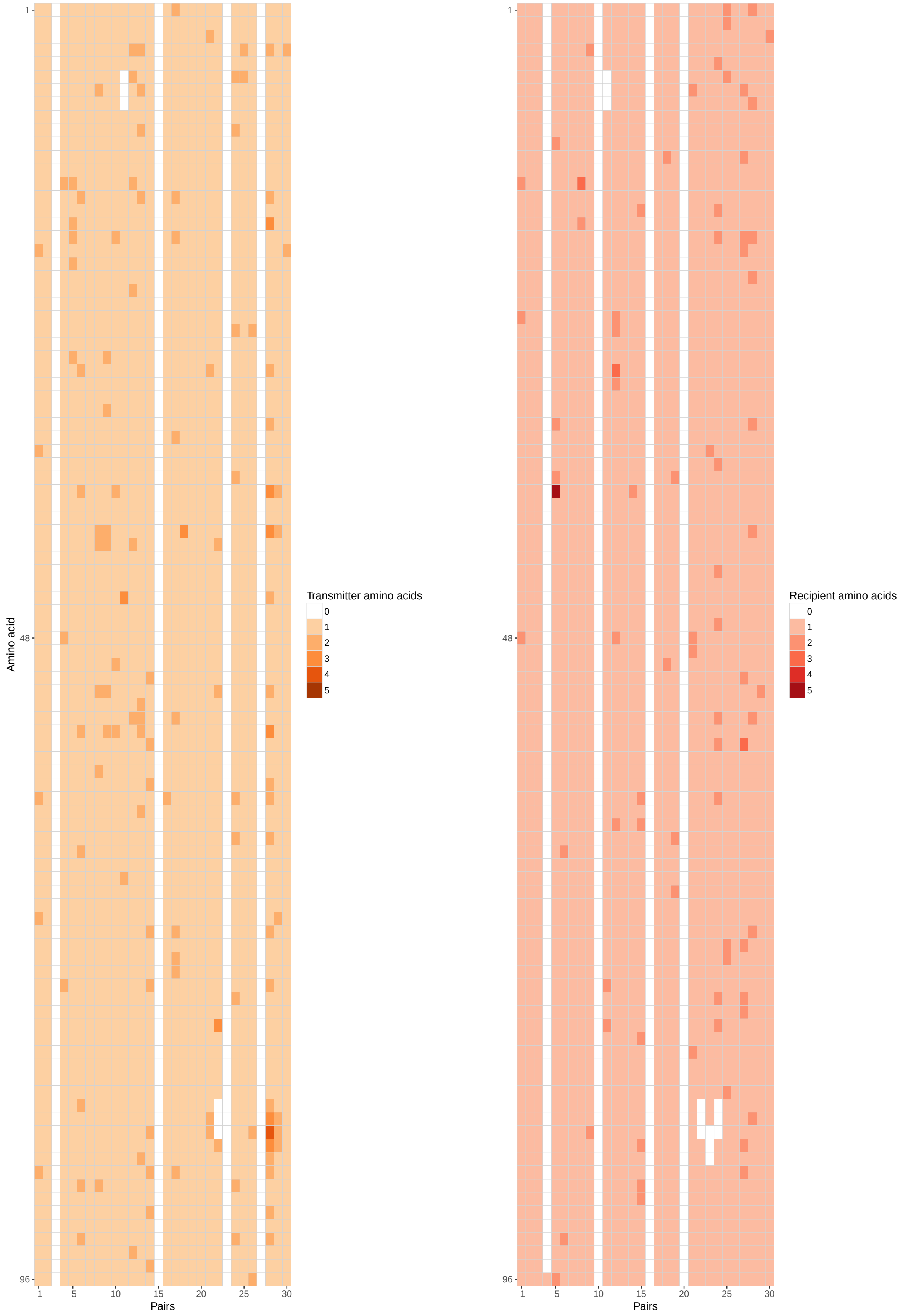

Figure S6-18. Heatmaps of individual amino acid counts for the *vpr* reading frame. The left respectively right heatmap shows the number of amino acids in the transmitter, respectively in the recipient at the analysed 96 positions across all 30 recipient and transmitters.

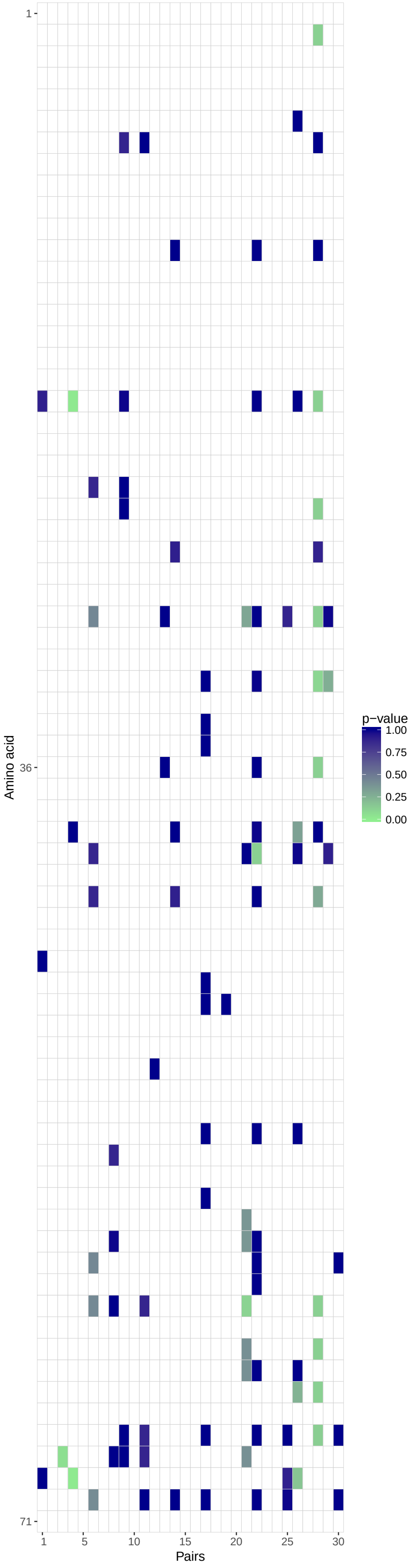

Figure S6-19. Heatmaps of p-values for the *tat* reading frame. Each column represents the test outcomes for a transmitter-recipient pair and every row represents one amino acid locus out of 71 across all 30 recipient and transmitters.

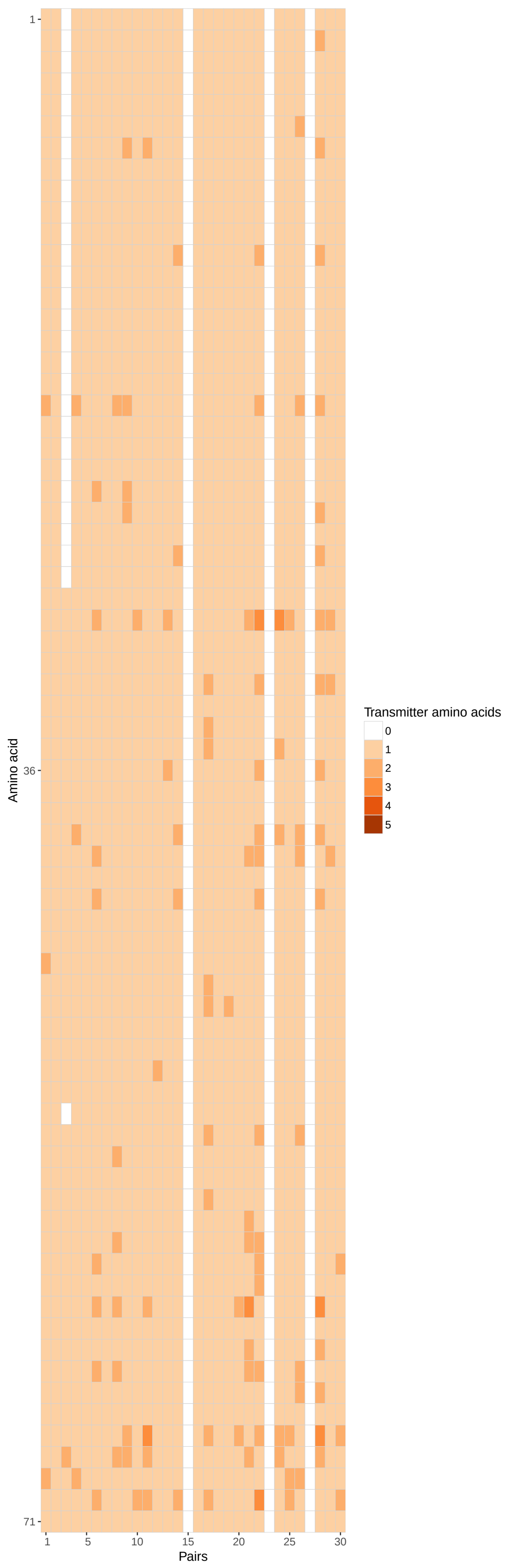

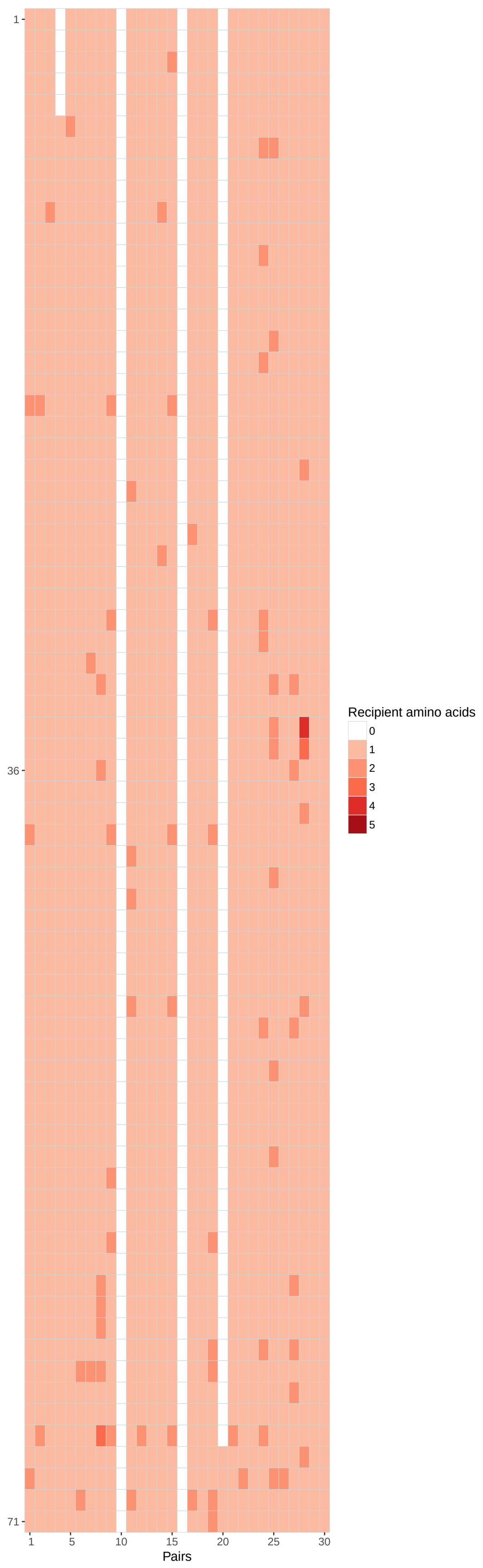

Figure S6-20. Heatmaps of individual amino acid counts for the *tat* reading frame. The left respectively right heatmap shows the number of amino acids in the transmitter, respectively in the recipient at the analysed 71 positions across all 30 recipient and transmitters.

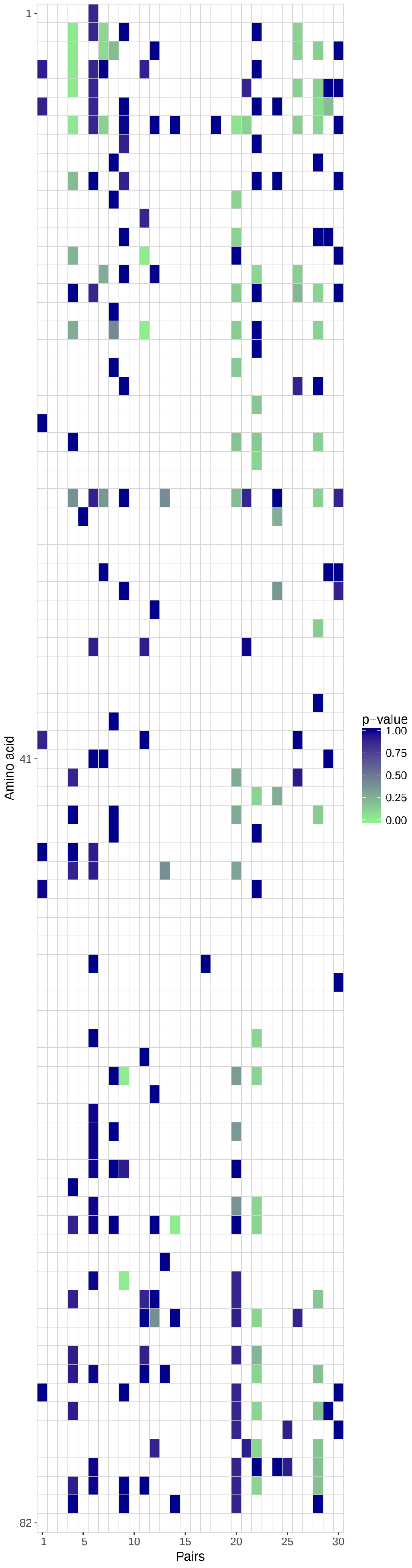

Figure S6-21. Heatmaps of p-values for the *vpu* reading frame. Each column represents the test outcomes for a transmitter-recipient pair and every row represents one amino acid locus out of 82 across all 30 recipient and transmitters.

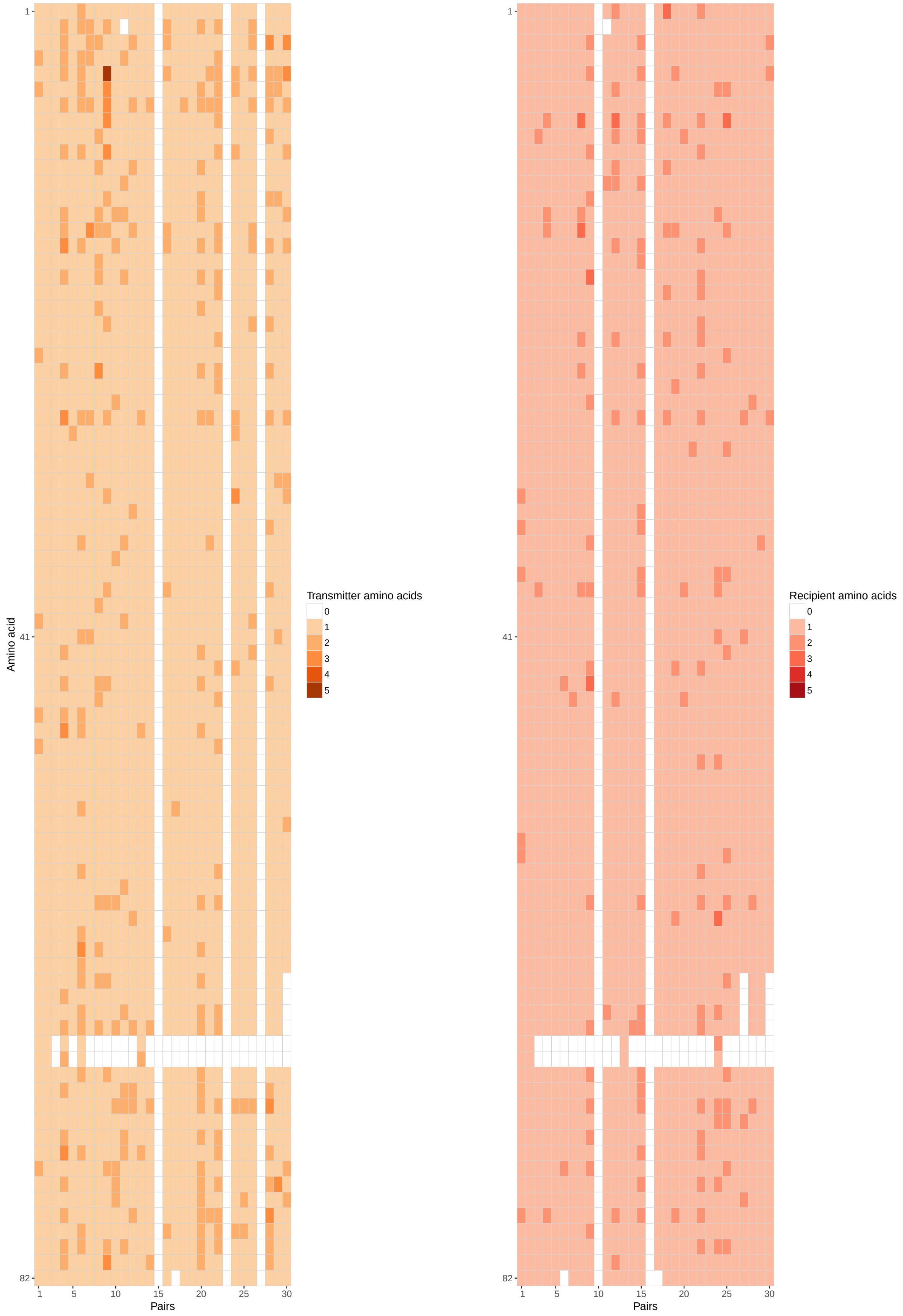

Figure S6-22. Heatmaps of individual amino acid counts for the *vpu* reading frame. The left respectively right heatmap shows the number of amino acids in the transmitter, respectively in the recipient at the analysed 82 positions across all 30 recipient and transmitters.

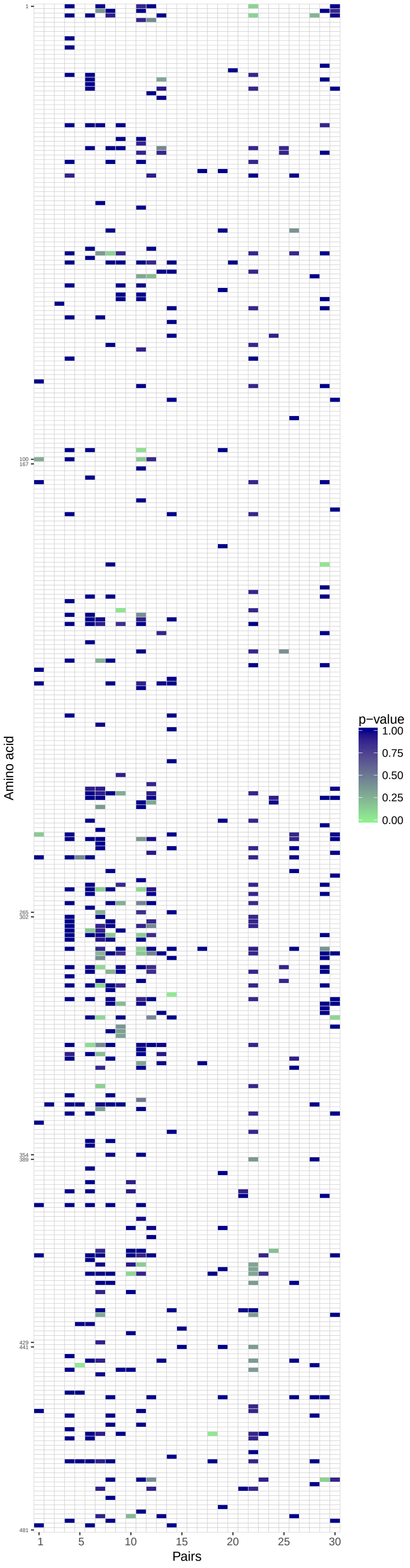

Figure S6-23. Heatmaps of p-values for the *gp120* reading frame. Each column represents the test outcomes for a transmitter-recipient pair and every row represents one amino acid locus out of 334 across all 30 recipient and transmitters.

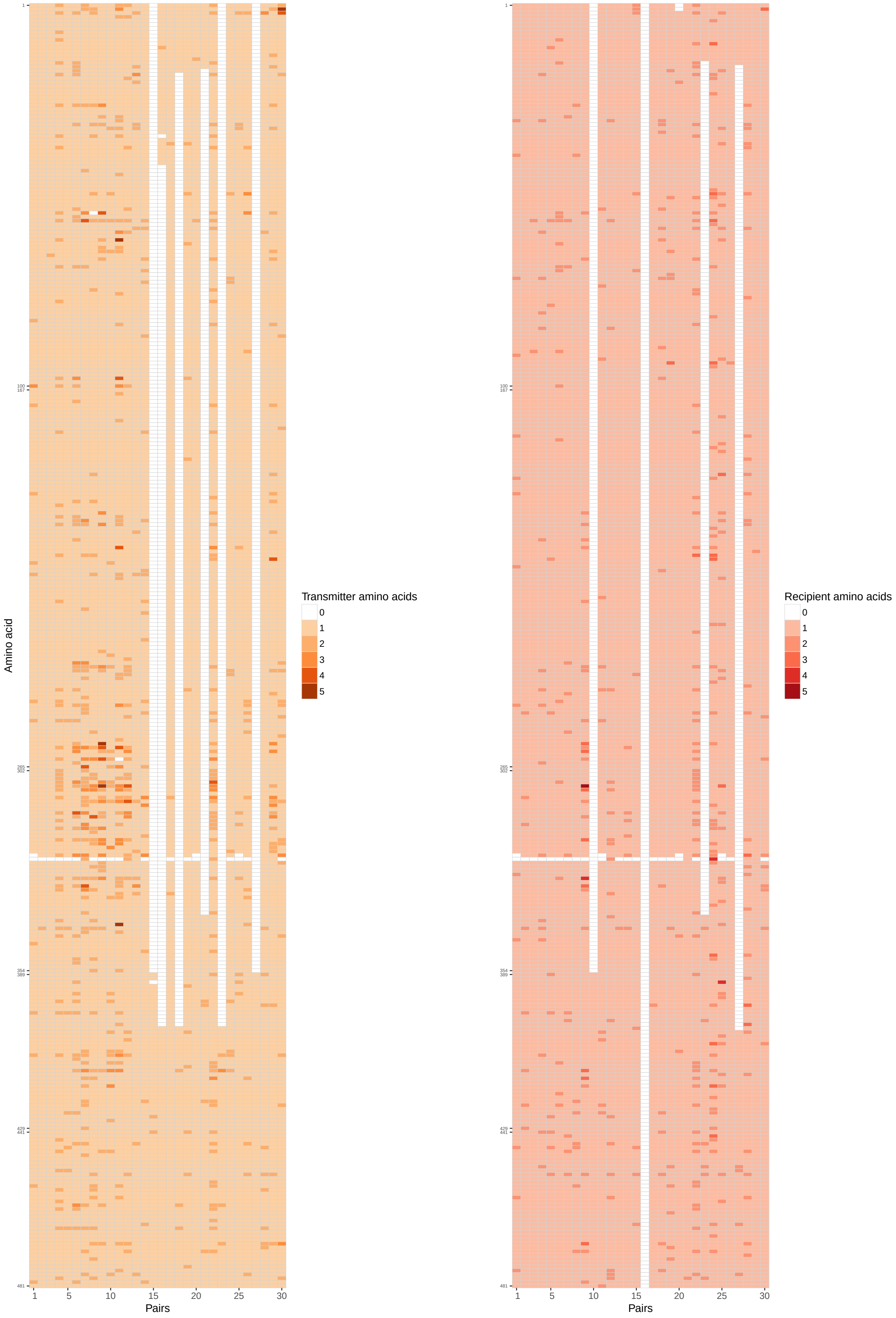

Figure S6-24. Heatmaps of individual amino acid counts for the *gp120* reading frame. The left respectively right heatmap shows the number of amino acids in the transmitter, respectively in the recipient at the analysed 334 positions across all 30 recipient and transmitters.

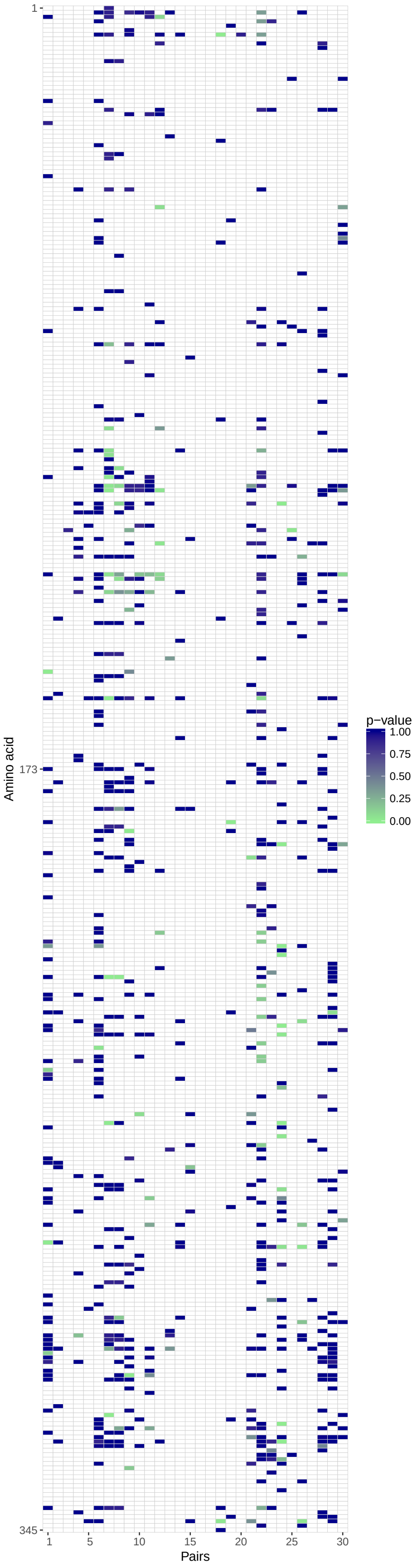

Figure S6-25. Heatmaps of p-values for the *gp41* reading frame. Each column represents the test outcomes for a transmitter-recipient pair and every row represents one amino acid locus out of 345 across all 30 recipient and transmitters.

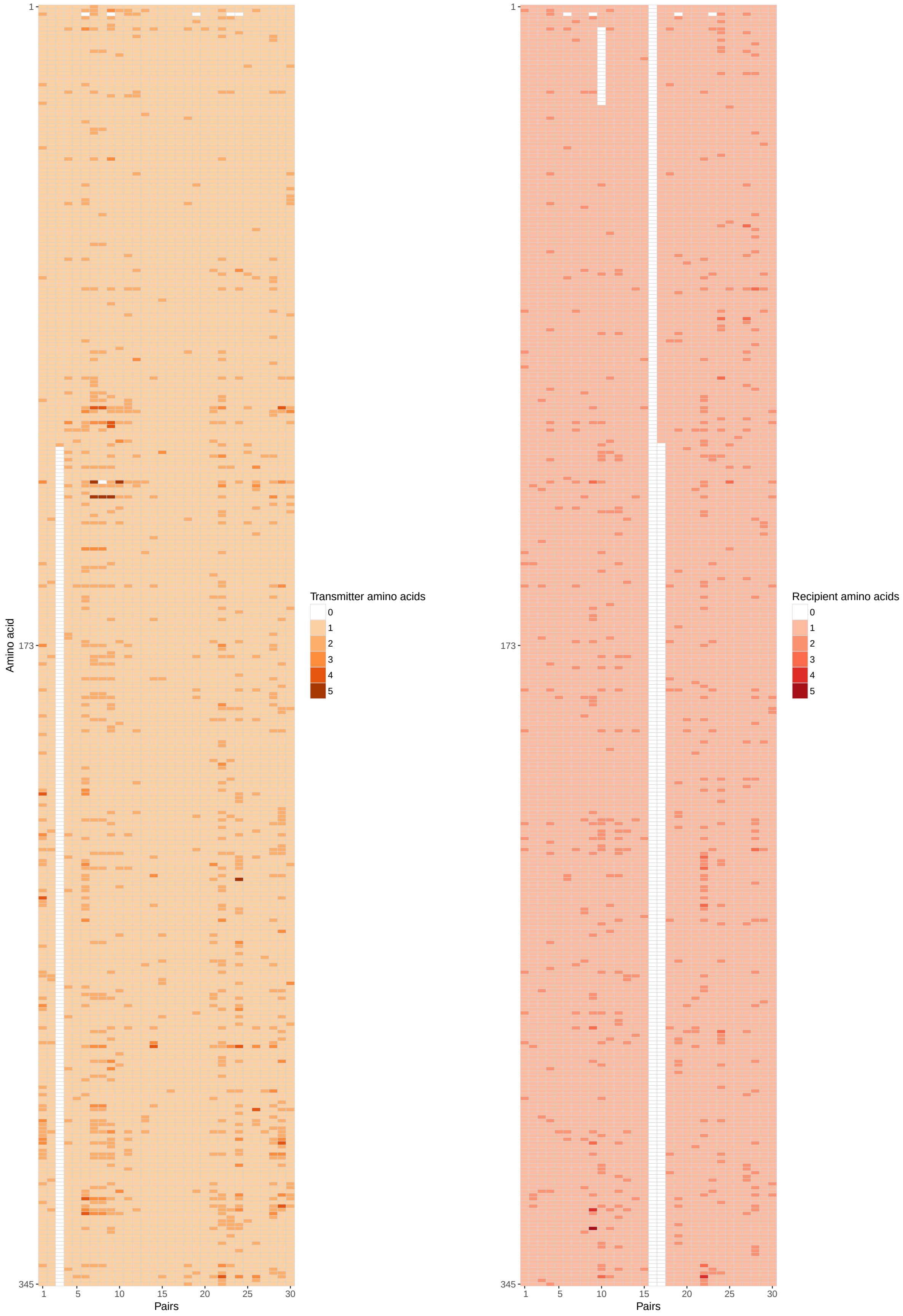

Figure S6-26. Heatmaps of individual amino acid counts for the *gp41* reading frame. The left respectively right heatmap shows the number of amino acids in the transmitter, respectively in the recipient at the analysed 345 positions across all 30 recipient and transmitters.

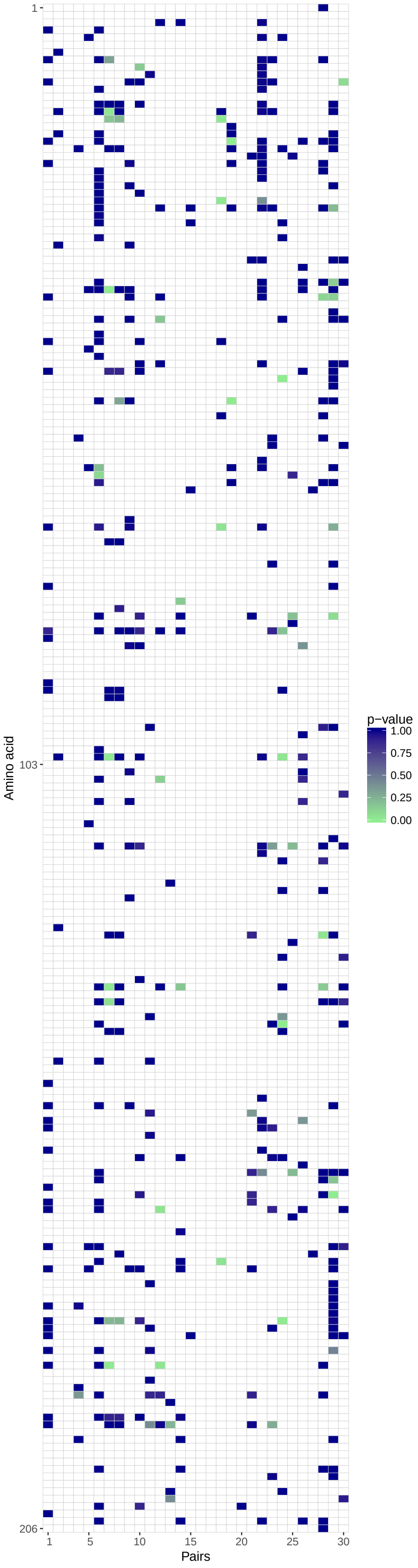

Figure S6-27. Heatmaps of p-values for the *nef* reading frame. Each column represents the test outcomes for a transmitter-recipient pair and every row represents one amino acid locus out of 206 across all 30 recipient and transmitters.

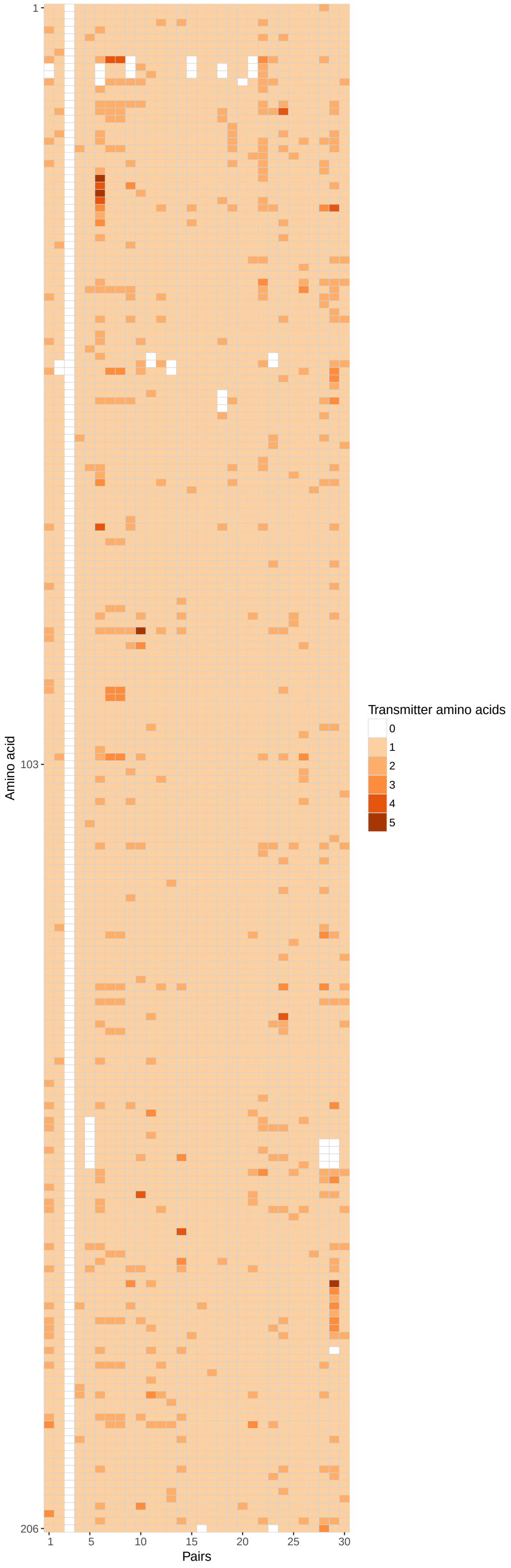

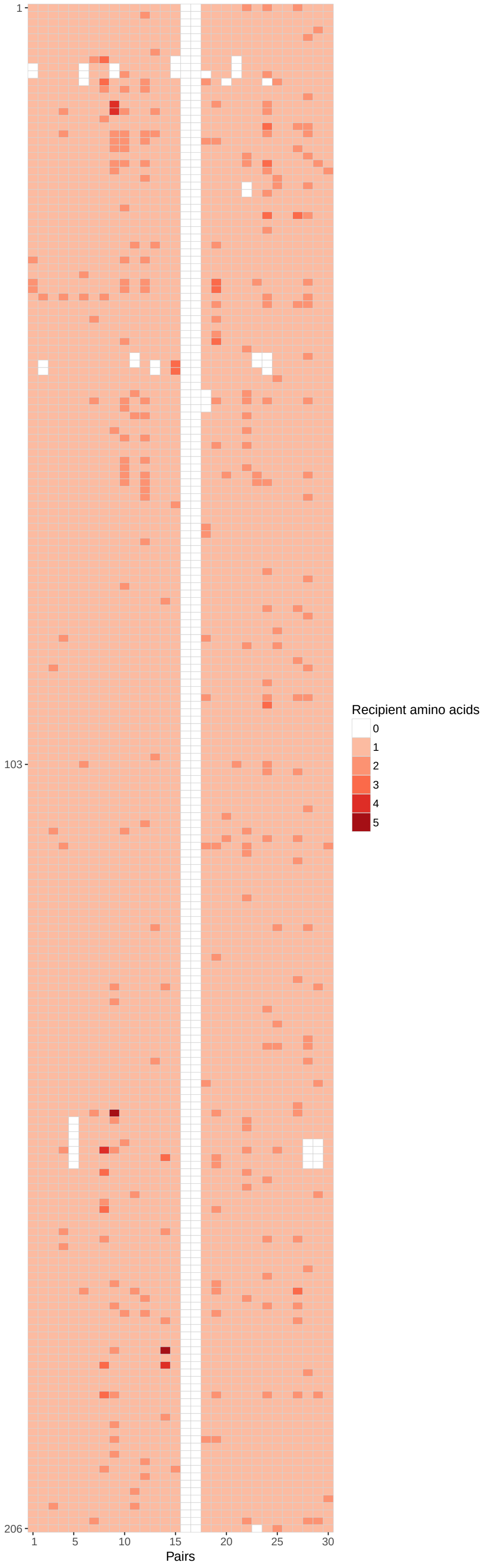

Figure S6-28. Heatmaps of individual amino acid counts for the *nef* reading frame. The left respectively right heatmap shows the number of amino acids in the transmitter, respectively in the recipient at the analysed 206 positions across all 30 recipient and transmitters.

References

Beijk, R., Mooibroek, D., van de Kassteele, J., and Hoogerbrugge, R. (2008). PM10: Equivalence study 2006. *RIVM Report*, **680708002**, 2008.

Blei, D. M., Ng, A. Y., and Jordan, M. I. (2003). Latent dirichlet allocation. *Journal of Machine Learning Research*, **3**, 993–1022.

Harrison, M. T. (2010). Conservative Hypothesis Tests and Confidence Intervals using Importance Sampling. *arXiv preprint arXiv:1004.2910*.

Keele, B. F., Giorgi, E. E., Salazar-Gonzalez, J. F., Decker, J. M., Pham, K. T., Salazar, M. G., Sun, C., Grayson, T., Wang, S., Li, H., *et al.* (2008). Identification and characterization of transmitted and early founder virus envelopes in primary HIV-1 infection. *Proceedings of the National Academy of Sciences*, **105**(21), 7552–7557.

Li, B., Gladden, A. D., Altfeld, M., Kaldor, J. M., Cooper, D. A., Kelleher, A. D., and Allen, T. M. (2007). Rapid reversion of sequence polymorphisms dominates early human immunodeficiency virus type 1 evolution. *Journal of Virology*, **81**(1), 193–201.

Nickle, D. C., Heath, L., Jensen, M. A., Gilbert, P. B., Mullins, J. I., and Pond, S. L. K. (2007). HIV-specific probabilistic models of protein evolution. *PLOS One*, **2**(6), e503.

Nowak, M. A. (2006). *Evolutionary Dynamics*. Harvard University Press.

Rugg, G. (2007). *Using Statistics: A Gentle Introduction*. McGraw-Hill International.
